# Structure‐Based Design and Biological Evaluation of Imidazo[1,2‐*a*]pyridine Derivatives as Potential Antituberculosis Agents

**DOI:** 10.1002/cbdv.71854

**Published:** 2026-10-22

**Authors:** Hanmant N. Chaugule, Shravan Y. Jadhav, Sujay A. Ghorpade, Dayanand C. Kore, Vaibhav R. Mane, Umakant M. Kadampalle, Vijay H. Masand, Magdi E. A. Zaki, Rohit S. Bavi, Samin A. Shaikh

**Affiliations:** ^1^ Department of Chemistry DBF Dayanand College of Arts and Science Solapur Maharashtra India; ^2^ Department of Chemistry Vidya Bharati Mahavidyalaya Amravati Maharashtra India; ^3^ Department of Chemistry, College of Science Imam Mohammad Ibn Saud Islamic University (IMSIU) Riyadh Saudi Arabia; ^4^ School of Chemical Sciences PAH Solapur University Solapur Maharashtra India; ^5^ Department of Chemistry, Kr. V. N. Naik Shikshan Prasarak Sanstha's Arts, Commerce and, Science College Savitribai Phule Pune University Nashik Maharashtra India

**Keywords:** antioxidant, antituberculosis, imidazopyridine, molecular docking, SAR

## Abstract

A library of 11 novel imidazopyridine‐based sulfonamide derivatives (**4a–4k**) were synthesized through the coupling of an imidazo[1,2‐*a*]pyridine hydrochloride intermediate with various substituted sulfonyl chloride derivatives (**3a–3k**). The structures of the compounds (**4a–4k**) were confirmed using appropriate spectroscopic techniques. The synthesized derivatives (**4a–4k**) were subsequently evaluated for their antitubercular and antioxidant potential. Among the tested molecules, compounds **4b**, **4d**, and **4g** demonstrated moderate antimycobacterial activity, displaying minimum inhibitory concentration (MIC) values of 6.21 ± 0.45 µg/mL, which were comparable to that of the standard drug rifampicin. To gain insight into their possible mechanism of action, molecular docking studies were carried out against selected tuberculosis (TB)‐associated protein targets. The active compounds exhibited favorable binding interactions within the target binding pockets, supporting the observed biological activity. Furthermore, *in silico* ADMET assessment revealed encouraging pharmacokinetic characteristics, including good gastrointestinal absorption, satisfactory bioavailability, and compliance with common drug‐likeness criteria without significant rule violations. Collectively, these findings identify the imidazopyridine sulfonamide derivatives as potential lead structures for further structural optimization and biological investigation toward the development of new antitubercular agents.

## Graphical abstract

A library of 11 novel imidazopyridine‐based sulfonamide derivatives (**4a–4k**) were synthesized through the coupling of an imidazo[1,2‐*a*]pyridine hydrochloride intermediate with various substituted arylsulfonyl chlorides (**3a–3k**). The synthesized derivatives (**4a–4k**) were subsequently evaluated for their antitubercular and antioxidant potential. To gain insight into their possible mechanism of action, molecular docking studies were carried out against selected tuberculosis‐associated protein targets. The active compounds exhibited favorable binding interactions within the target binding pockets, supporting the observed biological activity. Furthermore, *in silico* ADMET assessment revealed encouraging pharmacokinetic characteristics, including good gastrointestinal absorption, satisfactory bioavailability, and compliance with common drug‐likeness criteria without significant rule violations.

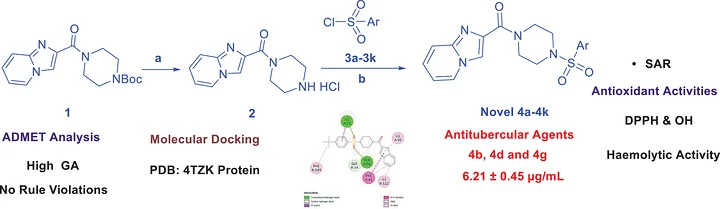

## 1 Introduction

Tuberculosis (TB) caused by *Mycobacterium tuberculosis*, continues to be a major global health challenge despite the availability of established treatment regimens. The increasing prevalence of multidrug‐resistant (MDR‐TB) and extensively drug‐resistant (XDR‐TB) strains has complicated disease management and reduced the effectiveness of conventional therapies [1]. In addition, lengthy treatment schedules, adverse drug reactions, and poor patient compliance further underscore the need for the development of novel antitubercular agents with improved therapeutic profiles and alternative mechanisms of action [2].

Among the diverse heterocyclic scaffolds explored in medicinal chemistry, Imidazo[1,2‐*a*]pyridines have attracted considerable attention because of their structural versatility and favorable pharmacological properties. Compounds containing this bicyclic framework have been reported to exhibit a wide range of biological activities, including anxiolytic [3], antimicrobial [4], antitubercular [5, 6], antiproliferative [7], anti‐inflammatory [8, 9] antioxidant [10, 11], and antidiabetic activities [12]. Owing to these attributes, the imidazo[1,2‐*a*]pyridine nucleus has emerged as an important platform for the design of biologically active molecules.

Likewise, the piperazine moiety represents a privileged structural motif frequently encountered in approved therapeutic agents. Incorporation of a piperazine ring often improves physicochemical and pharmacokinetic properties, particularly aqueous solubility and target‐binding capability. Consequently, piperazine‐containing compounds have demonstrated diverse pharmacological activities, including antibacterial [13], anti‐inflammatory [14], antioxidant [15], anticancer [16], antidiabetic [17], anti‐Alzheimer [18], antiviral [19], anti‐HIV [20], antitumor [21], antifungal [22], antimicrobial [23], antiproliferative agents [24], and antitumor agents [25].

Sulfonamide‐containing molecules also occupy a prominent position in drug discovery. The sulfonyl group is capable of participating in favorable hydrogen‐bonding and electrostatic interactions within biological targets, thereby enhancing molecular recognition and binding affinity [26, 27, 28, 29]. Combining imidazo[1,2‐*a*]pyridine, piperazine, and sulfonyl pharmacophores within a single molecular architecture may therefore provide a promising strategy for generating compounds with enhanced antimycobacterial potential. Representative bioactive molecules containing pyridine–imidazole and sulfonyl‐linked structural features are presented in Figure 1. Despite extensive investigation of imidazo[1,2‐*a*]pyridine derivatives, reports describing piperazine‐linked sulfonamide analogues as antitubercular agents remain limited.

**FIGURE 1:**
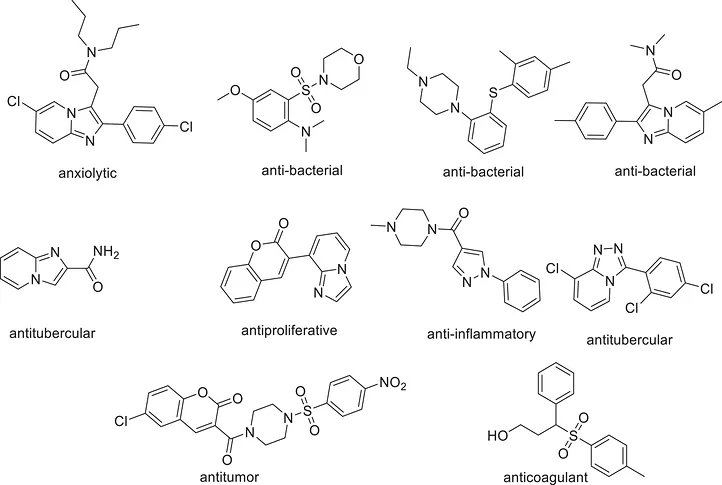
Representative bioactive compounds containing pyridine–imidazole and sulfonyl‐linked structural moieties.

Recent antitubercular drug‐discovery studies further demonstrate the continuing importance of heterocyclic molecular design and structure‐based approaches. For example, recent triazole–quinazolinone hybrids were evaluated against *M. tuberculosis* and investigated by molecular docking, demonstrating the value of heterocyclic hybridization in the search for new antitubercular chemotypes [30]. Similarly, indanol–1,2,3‐triazole derivatives have been reported as antitubercular and antimicrobial agents, with selected compounds showing activity against *M. tuberculosis* and further evaluation through molecular docking against InhA and *in silico* ADME profiling [31]. These studies illustrate the contemporary use of heterocyclic diversification, molecular hybridization, biological screening, molecular docking, and pharmacokinetic prediction in antitubercular medicinal‐chemistry research.

During *tuberculosis* infection, macrophage‐mediated immune responses trigger the production of reactive oxygen and nitrogen species (ROS/RNS) as part of the host defense mechanism against intracellular bacilli [32]. Although these species contribute to pathogen elimination, excessive ROS/RNS generation may induce oxidative stress, leading to cellular damage through oxidation of lipids, proteins, and nucleic acids. Therefore, evaluation of antioxidant properties may provide additional information regarding the potential of candidate molecules to alleviate oxidative stress associated with infection. In the present investigation, antioxidant activity was assessed as a complementary biological parameter alongside antitubercular screening. Nevertheless, such activity should be interpreted as an auxiliary pharmacological characteristic rather than a direct indicator of antimycobacterial efficacy.

Besides biological activity, early evaluation of pharmacokinetic behavior and safety‐related parameters is essential during lead identification and optimization [33]. Computational ADMET analysis has become a valuable approach for predicting absorption, distribution, metabolism, excretion, toxicity, oral bioavailability, and overall drug‐likeness, thereby facilitating the selection of promising candidates for further development [34]. Motivated by these considerations, a series of 11 novel imidazo[1,2‐*a*]pyridine‐based sulfonamide derivatives were designed and synthesized through the reaction of imidazo[1,2‐*a*]pyridin‐2‐yl(piperazin‐1‐yl)methanone hydrochloride with various sulfonyl chloride derivatives. The synthesized compounds (**4a–4k**) were evaluated for their antitubercular activity against the H37Ra strain of *M. tuberculosis*. In addition, antioxidant potential and cytotoxicity were investigated to obtain a broader assessment of their biological profile. Molecular docking studies were conducted to explore the binding interactions of the synthesized derivatives with selected protein targets and to provide structural insights into their observed activity.

Furthermore, SwissADME‐based *in silico* analyses were performed to assess the pharmacokinetic properties, drug‐likeness and developability of the newly synthesized compounds.

## 2 Results and Discussion

### 2.1 Synthetic Chemistry

The synthetic route employed for the preparation of the target imidazo[1,2‐*a*]pyridine derivatives (**4a–4k**) is illustrated in Scheme 1. The synthesis commenced from *tert*‐butyl 4‐(imidazo[1,2‐*a*]pyridine‐2‐carbonyl)piperazine‐1‐carboxylate (**1**) which served as the key intermediate for subsequent functionalization.

**SCHEME 1:**
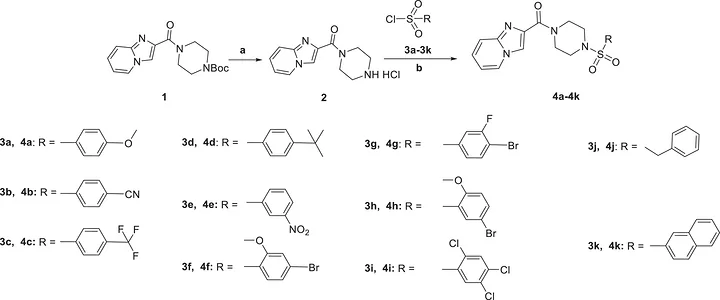
Synthesis of novel imidazopyridine derivatives **(4a–4k)**. Reaction conditions: (a) HCl (40 mmoles), 1,4‐dioxane; (b) sulfonyl chloride derivatives (**3a–3k**) (11 mmoles), DIPEA (30 mmoles), DMF.

Initially, deprotection of compound (**1**) was achieved by treatment with hydrochloric acid, affording imidazo[1,2‐*a*]pyridin‐2‐yl(piperazin‐1‐yl)methanone hydrochloride (**2**) in good yield. The resulting intermediate was subsequently reacted with a series of sulfonyl chloride derivatives (**3a–3k**) in dimethylformamide (DMF) under appropriate basic conditions to furnish the desired sulfonamide‐linked imidazo[1,2‐*a*]pyridine derivatives (**4a–4k**).

The adopted synthetic protocol proved to be efficient and versatile, enabling the preparation of structurally diverse analogues in satisfactory yields ranging from 60% to 85%. The reactions generally reached completion within 12–14 h, and the target compounds were isolated with minimal purification requirements. The straightforward reaction sequence and broad substrate compatibility make this approach suitable for the rapid generation of imidazo[1,2‐*a*]pyridine‐based molecular libraries for biological evaluation.

### 2.2 Spectral Analysis

The structures of the synthesized imidazo[1,2‐*a*]pyridine derivatives (**4a–4k**) were established using a combination of ^1^H‐NMR, ^13^C‐NMR, IR, and mass spectral analysis which collectively confirmed the successful formation of the target molecules. The signals associated with the heteroaromatic imidazo[1,2‐*a*]pyridine framework appeared within the range of 1500–1600 cm^−^^1^. The presence of the sulfonamide functionality was evidenced by strong absorption bands attributed to the asymmetric stretching of the SO_2_ group, typically observed between 1320 cm^−^^1^ and 1355 cm^−^^1^.

The ^1^H‐NMR spectra confirmed the aromatic protons in the range of chemical shift 6.5–8.5 ppm in all compounds **4a–4k**, while methoxy groups were confirmed at 3.5–4.0 ppm in compounds **4a**, **4f**, and **4h**. The characteristic imidazo[1,2‐*a*]pyridine proton appeared as a singlet at 8.0 ppm in compounds **4a**–**4k**. The ^13^C NMR spectra displayed the expected carbon resonances for the synthesized derivatives, including the characteristic carbonyl carbon signal in the corresponding downfield region, while aromatic and heteroaromatic carbons appeared mainly within the δ 110–160 ppm range. The methoxy‐substituted derivatives **4a**, **4f**, and **4h** additionally showed OCH_3_ carbon resonances in the expected aliphatic region.

Furthermore, the mass spectra of all compounds (**4a–4k**) showed peaks [M]^+^, [M + 1]^+^/[M + 2]^+^ that matched the calculated exact masses. The compound **4a** calculated *m*/*z* at 400.1205 [M]^+^ and peak observed at 401.1271 [M + 1]^+^ in mass spectrum. The compound **4f** showed a prominent ion peak at *m*/*z* 479.3490 [M + 1]^+^ matched with calculated *m*/*z* at 478.0310 [M]^+^.

The synthesized imidazo[1,2‐*a*]pyridine derivatives were characterized by FT‐IR, ^1^H NMR, and ^13^C NMR spectroscopy. Furthermore, high‐resolution mass spectrometric (HRMS) data were obtained for the compounds for which experimental HRMS measurements were available. The observed molecular ion peaks were consistent with the calculated exact masses and supported the proposed molecular formulas. (Representative HRMS spectra are provided in the Supporting Information. The complete spectral data and copies of all available spectra are provided in Figures S1–S43).

### 2.3 Biological Activities

#### 2.3.1 Antitubercular Activities

The synthesized imidazo[1,2‐*a*]pyridine derivatives (**4a–4k**) were evaluated for their antimycobacterial activity against *M. tuberculosis* H37Ra using the microplate Alamar Blue assay (MABA). The obtained MIC values are presented in Table 1. All compounds exhibited detectable inhibitory activity against the tested strain, with MIC values ranging from 6.21 ± 0.45 to 8.55 ± 0.45 µg/mL, demonstrating that the incorporation of sulfonamide moieties into the imidazo[1,2‐*a*]pyridine framework is favorable for antitubercular activity.

**TABLE 1:** MIC of compounds **(4a–4k)** against *Mycobacterium tuberculosis* H37Ra.

Comp	MIC (µg/mL)
**4a**	7.73 ± 0.22
**4b**	**6.21 ± 0.45**
**4c**	8.05 ± 0.36
**4d**	**6.21 ± 0.45**
**4e**	**6.50 ± 0.72**
**4f**	7.69 ± 0.22
**4g**	**6.21 ± 0.45**
**4h**	7.92 ± 0.22
**4i**	6.99 ± 0.22
**4j**	8.55 ± 0.45
**4k**	**6.48 ± 0.22**
**Rifampicin**	**5.2 ± 0.22**

*Note*: Three independent experiments (*n* = 3), mean ± SD.

Among the evaluated derivatives, compounds **4b**, **4d**, and **4g** emerged as the most active members of the series, each displaying an MIC value of 6.21 ± 0.45 µg/mL. These values are close to that observed for the reference drug rifampicin (MIC = 5.20 ± 0.22 µg/mL), indicating that these derivatives possess promising antimycobacterial potential. Compounds **4e** and **4k** also exhibited notable activity, with MIC values of 6.50 ± 0.72 and 6.48 ± 0.22 µg/mL, respectively, suggesting that substitution on the sulfonyl ring plays an important role in modulating biological activity.

In contrast, compounds **4c**, **4h**, and **4j** demonstrated comparatively lower potency, exhibiting MIC values of 8.05 ± 0.36, 7.92 ± 0.22, and 8.55 ± 0.45 µg/mL, respectively. The observed variation in activity within the series indicates that both the electronic nature and positional arrangement of substituents influence the interaction of the molecules with mycobacterial targets.

Nevertheless, the relatively narrow MIC range across all analogues suggests that the imidazo[1,2‐*a*]pyridine–piperazine–sulfonamide scaffold provides a favorable structural framework for antimycobacterial activity.

These biological data identify compounds **4b**, **4d**, and **4g** exhibited comparatively better antitubercular activity within the present series and may serve as potential lead structures requiring further optimization and experimental validation.

#### 2.3.2 Antioxidant Activity

The antioxidant properties of the synthesized imidazo[1,2‐*a*]pyridine derivatives (**4a–4k**) were investigated using DPPH radical scavenging and hydroxyl radical (•OH) scavenging assays. The results presented in Table 2 indicate that all compounds exhibited measurable antioxidant activity, although their efficacy varied depending on the nature of the substituent present on the sulfonyl moiety.

**TABLE 2:** Antioxidant activities of compounds **(4a–4k)**.

Comp	DPPH scavenging (%)	Hydroxyl radical scavenging (%)
**4a**	70.85	71.52
**4b**	65.17	68.18
**4c**	59.41	68.15
**4d**	71.02	63.25
**4e**	72.16	64.05
**4f**	61.68	63.25
**4g**	64.02	68.15
**4h**	52.15	54.15
**4i**	69.25	64.05
**4j**	67.15	68.14
**4k**	69.25	66.84
**Ascorbic acid**	81.25	NA
**α‐Tocopherol**	NA	83.15

*Note*: NA, not applicable. Values represent mean values from three independent experiments (*n* = 3).

In the DPPH assay, the tested compounds displayed radical scavenging activities ranging from 52.15% to 72.16%. Among the series, compound **4e** demonstrated the highest activity (72.16%), followed closely by compounds **4d** (71.02%) and **4a** (70.85%). Although these values were lower than that observed for the reference antioxidant ascorbic acid (81.25%), several derivatives exhibited appreciable free‐radical scavenging capacity, suggesting that the imidazo[1,2‐*a*]pyridine scaffold contributes positively to antioxidant behavior.

Evaluation of hydroxyl radical scavenging activity revealed a similar trend, with percentage inhibition values ranging from 54.15% to 71.52%. Compound **4a** emerged as the most active derivative in this assay, exhibiting 71.52% scavenging activity, followed by compounds **4b** (68.18%), **4c** (68.15%), **4g** (68.15%), and **4j** (68.14%). Although the activities remained lower than that of the standard α‐tocopherol (83.15%), several compounds displayed moderate to good hydroxyl radical scavenging potential.

The observed antioxidant activity may be attributed to the presence of electron‐donating and ‐withdrawing substituents capable of stabilizing radical intermediates through resonance and inductive effects. However, no direct correlation between antioxidant activity and antitubercular potency was evident within the present series. Therefore, the antioxidant results should be regarded as a complementary pharmacological property that may contribute to reducing oxidative stress associated with tuberculosis infection rather than as a primary determinant of antimycobacterial activity.

#### 2.3.3 Hemolytic Activity

The hemolytic potential of the synthesized imidazo[1,2‐*a*]pyridine derivatives (**4a–4k**) was evaluated using human erythrocytes as a preliminary assessment of membrane toxicity. The obtained results are summarized in Table 3. All tested compounds exhibited very low levels of hemolysis, with percentage hemolysis values ranging from 1.05 ± 0.020% to 2.90 ± 0.020%, indicating minimal disruption of erythrocyte membrane integrity under the experimental conditions.

**TABLE 3:** Hemolytic activity of compounds **(4a–4k)**.

Comp	% Hemolysis
**4a**	1.30 ± 0.005
**4b**	1.70 ± 0.005
**4c**	2.04 ± 0.030
**4d**	1.88 ± 0.005
**4e**	2.90 ± 0.020
**4f**	1.59 ± 0.020
**4g**	2.06 ± 0.020
**4h**	2.66 ± 0.020
**4i**	1.37 ± 0.025
**4j**	1.95 ± 0.026
**4k**	1.05 ± 0.020
**Triton X 100**	11.26 ± 0.971

*Note*: *n* = 3, mean ± SD.

Among the evaluated derivatives, compound **4e** displayed the highest hemolytic activity (2.90 ± 0.020%), whereas compound **4k** showed the lowest value (1.05 ± 0.020%). Importantly, none of the synthesized compounds exceeded the commonly accepted threshold of 5% hemolysis for preliminary biocompatibility assessment, suggesting a favorable safety profile. In contrast, the positive control Triton X‐100 produced substantially higher hemolysis (11.26 ± 0.971%), confirming the reliability and responsiveness of the assay system.

The low hemolytic activity observed across the series indicates that incorporation of the imidazo[1,2‐*a*]pyridine–piperazine–sulfonamide framework does not significantly compromise erythrocyte membrane stability. Furthermore, the active antitubercular derivatives, including compounds **4b**, **4d**, and **4g**, exhibited only minimal hemolytic effects, suggesting a potentially advantageous balance between biological activity and preliminary safety. Nevertheless, additional toxicological investigations involving dose‐dependent studies and mammalian cell‐based assays will be required to fully establish the safety profile of these compounds.

### 2.4 Structure–Activity Relationship (SAR) Analysis

The antitubercular activity of the synthesized imidazo[1,2‐*a*]pyridine sulfonamide derivatives (**4a–4k**) was influenced by the nature, electronic characteristics, steric requirements, hydrophobicity, and conformational flexibility of the substituent attached to the sulfonyl group (Table 1). The compounds displayed MIC values ranging from 6.21 ± 0.45 µg/mL to 8.55 ± 0.45 µg/mL, indicating a relatively narrow activity range across the series. This observation suggests that the common imidazo[1,2‐*a*]pyridine–piperazine–sulfonamide framework contributes substantially to the antimycobacterial profile, while modifications of the sulfonyl‐linked substituent modulate activity rather than completely altering it.

Among the synthesized derivatives, **4b** (*p*‐CN), **4d** (*p*‐*tert*‐butyl), and **4g** (*m*‐F, *p*‐Br) exhibited the highest activity, with MIC values of 6.21 ± 0.45 µg/mL. The activity profile of these compounds indicates that both electronic and hydrophobic factors can contribute to activity, but neither property alone appears sufficient to explain the observed SAR.

The *p*‐cyano derivative **4b** showed better activity than the *p*‐CF_3_ derivative **4c**, despite both substituents being strongly electron‐withdrawing. This comparison indicates that the electron‐withdrawing strength of the substituent is not the sole determinant of activity. The cyano group is smaller and more polar than CF_3_ and introduces a localized nitrile functionality without substantially increasing steric bulk. Consequently, **4b** may provide a more favorable balance between electronic distribution, polarity, steric demand, and hydrophobicity than **4c**. In contrast, the greater size and hydrophobic character of the CF_3_ group may influence the orientation and spatial accommodation of the sulfonyl moiety within the relevant biological environment. Thus, the higher activity of **4b** suggests that substituent size and polarity, in addition to electron‐withdrawing character, are important determinants of activity.

The activity of **4d** containing a *para*‐*tert*‐butyl group provides further evidence that strong electron withdrawal is not essential for favorable activity. The *tert*‐butyl substituent substantially increases the hydrophobic surface of the molecule and may facilitate favorable hydrophobic and van der Waals contacts within nonpolar regions of the biological target. Its *para* substitution also provides a relatively extended substituent while maintaining a defined spatial relationship with the sulfonyl group. The favorable activity of **4d** therefore suggests that appropriate hydrophobic surface area and steric occupancy can compensate for the absence of a strongly electron‐withdrawing substituent. Nevertheless, the present experimental data do not establish a direct interaction between the *tert*‐butyl group and a specific protein residue.

The dihalogenated derivative **4g** (*m*‐F, *p*‐Br) also exhibited an MIC of 6.21 ± 0.45 µg/mL. The combined presence of fluorine and bromine provides a distinct electronic and steric environment and increases the polarizability and hydrophobic surface of the aryl ring. The relatively small fluorine atom can modify the electronic character of the aromatic system, whereas the larger bromine atom may influence the three‐dimensional orientation and steric occupancy of the substituent. The high activity of **4g** therefore suggests that an appropriate combination of electronic modulation, polarizability, hydrophobicity, and steric complementarity may be beneficial for this scaffold.

Derivative **4e** (*m*‐NO_2_) also displayed comparatively good activity, with an MIC of 6.50 ± 0.72 µg/mL. The nitro group is strongly electron‐withdrawing and substantially increases the polarity of the aryl substituent. Its favorable activity suggests that an appropriately positioned polar electron‐withdrawing group can be accommodated within the molecular framework. However, comparison with **4b** and **4c** indicates that the biological response cannot be explained solely by electron‐withdrawing ability, highlighting the importance of the overall physicochemical and three‐dimensional properties of each derivative.

The naphthyl derivative **4k** retained comparatively good antitubercular activity, with an MIC of 6.48 ± 0.22 µg/mL, despite possessing a larger fused aromatic system. The naphthalene ring provides an extended hydrophobic and π‐surface that may favor van der Waals and hydrophobic interactions with complementary regions of the biological target. The retention of activity suggests that the relevant binding environment can accommodate a relatively bulky aromatic substituent when its spatial orientation is favorable. Interestingly, **4k** exhibited the most favorable predicted docking energy in the series, although its experimental MIC was not the lowest. This observation indicates that docking score alone cannot be used as a direct predictor of experimental antitubercular potency.

In contrast, the benzyl derivative **4j** exhibited the weakest activity, with an MIC of 8.55 ± 0.45 µg/mL. Although the benzyl group provides additional hydrophobic surface, it contains an intervening methylene group that increases conformational flexibility and changes the spatial relationship between the aromatic ring and the sulfonyl moiety. This flexibility may reduce the ability of the molecule to maintain an optimal orientation within the relevant biological environment and may consequently decrease favorable molecular recognition. The lower activity of 4j therefore suggests that hydrophobicity alone is insufficient and that the spatial presentation and conformational properties of the substituent are also important.

The methoxy‐containing derivatives **4a** (*p*‐OCH_3_), **4f** (*o*‐OCH_3_, *p*‐Br), and **4h** (*m*‐OCH_3_, *m*‐Br) showed comparatively lower activity, with MIC values within the range of approximately 7.69–7.92 µg/mL. The electron‐donating character of methoxy substitution, together with its positional and steric effects, may alter the electronic distribution and preferred orientation of the sulfonyl moiety. The reduced activity of these derivatives compared with **4b**, **4d**, and **4g** suggests that the introduction of methoxy groups does not provide the optimal balance of electronic and steric properties required for improved antimycobacterial activity in this series.

Overall, the SAR analysis indicates that the antitubercular activity of compounds **4a–4k** is governed by a multifactorial balance of electronic properties, hydrophobic surface area, steric demand, conformational flexibility, polarity, and three‐dimensional substituent orientation rather than by a simple electron‐withdrawing or hydrophobicity‐based relationship. The most active derivatives, **4b**, **4d**, and **4g**, represent structurally distinct substitution patterns, further supporting the importance of achieving an appropriate physicochemical and spatial balance. Molecular docking provides additional structural context for the observed trends; however, the lack of a strict correlation between docking scores and experimental MIC values emphasizes that docking predictions should be interpreted as supportive rather than definitive evidence of biological potency. Further systematic modification of the sulfonyl‐linked substituent and experimental target‐validation studies will be required to establish a more definitive SAR and guide the development of these compounds as potential lead structures requiring further optimization and biological investigation.

### 2.5 Molecular Docking

#### 2.5.1 Target Selection, Docking Protocol, and Interaction Analysis

Molecular docking was performed to investigate the possible binding modes of the synthesized imidazo[1,2‐*a*]pyridine sulfonamide derivatives within a therapeutically relevant *M. tuberculosis* target. Among the available tuberculosis‐associated targets, enoyl‐[acyl‐carrier‐protein] reductase (InhA) was selected because of its established role in mycobacterial fatty‐acid metabolism and mycolic‐acid biosynthesis, processes that are essential for the integrity and survival of the mycobacterial cell envelope. InhA is therefore a well‐established target for antitubercular drug discovery.

The crystal structure PDB: 4TZK was selected because it provides a high‐resolution (1.62 Å) x‐ray structure of *M. tuberculosis* InhA in complex with a co‐crystallized inhibitor and the NAD cofactor. The experimentally defined ligand‐binding environment provides a structurally characterized template for evaluating the accommodation and interaction patterns of the newly synthesized compounds.

The selection of InhA was also considered in the context of recent antitubercular drug‐discovery studies employing structure‐based approaches against validated mycobacterial targets. Recent investigations have explored different chemical scaffolds using molecular docking against tuberculosis‐associated targets, demonstrating the utility of structure‐based analysis for rationalizing ligand–target interactions [35, 36].

In the present study, 4TZK was therefore selected primarily on the basis of biological relevance of InhA, availability of a high‐resolution experimentally determined structure, presence of a defined inhibitor‐binding site, and suitability for structure‐based analysis, rather than on the basis of the docking scores obtained for the synthesized compounds.

The protein structure was prepared prior to docking, and the synthesized ligands were subjected to energy minimization before docking. The resulting poses were analyzed to identify favorable ligand orientations and possible non‐covalent interactions within the InhA‐binding cavity. The docking results were interpreted as a structural complement to the experimental antitubercular data rather than as a direct quantitative prediction of MIC values.

The docking calculations showed binding‐energy values ranging from –6.7 kcal/mol to –9.0 kcal/mol for the investigated derivatives (Table 4). Although these predicted energies should not be interpreted as direct measures of experimental inhibitory potency, the docking analysis was used primarily to examine the relative binding orientations and molecular interactions of the synthesized compounds within the InhA‐binding pocket.

**TABLE 4:** Docking analysis of compounds **(4a–4k)**.

Comp	Binding energy (kcal/mol)	H‐Bond for 4TZK
**4a**	−7.5	Gly96, Ser94
**4b**	−8.2	Thr17, Gly96, and Ser94
**4c**	−7.7	—
**4d**	−7.7	Ile21, Ser94
**4e**	−7.0	Thr17, Ser20
**4f**	−6.7	—
**4g**	−7.8	Gly96, Ser94
**4h**	−6.9	—
**4i**	−7.1	—
**4j**	−7.2	—
**4k**	−9.0	Lys165, Gly96

Among the evaluated derivatives, compound **4k** exhibited the most favorable predicted binding energy (–9.0 kcal/mol), whereas compounds **4b** and **4g** showed binding energies of –8.2 and –7.8 kcal/mol, respectively. Compounds **4d**, **4e**, and **4a** also adopted favorable binding orientations within the active site. The docking poses indicated that the synthesized derivatives could be accommodated within the hydrophobic binding cavity and establish hydrogen‐bonding and other non‐covalent interactions with residues lining the binding site.

Importantly, the docking results were considered together with the experimental antitubercular data rather than being used as an independent measure of potency. For example, compounds **4b**, **4d**, and **4g** showed the most promising experimental activity, while **4k** exhibited the lowest predicted docking energy. This lack of a strict rank‐order correlation indicates that factors beyond predicted binding energy, including ligand permeability, intracellular exposure, target accessibility, and other pharmacokinetic properties, may contribute to the observed biological activity.

Thus, the primary purpose of the docking study was to provide a structural rationale for the potential interaction of the synthesized compounds with InhA and to identify favorable binding modes that may assist further structural optimization of this scaffold.

It should be emphasized that the calculated docking energies were used primarily for comparative assessment of ligand accommodation and interaction patterns and should not be interpreted as direct measures of experimental antitubercular potency. Indeed, the compound displaying the most favorable predicted docking score was not identical to all compounds showing the lowest experimental MIC values, indicating that factors beyond calculated binding energy may contribute to the observed biological response.

#### 2.5.2 Docking Results and Interaction Analysis

The ligands were found to be interacting in various non‐bonded interactions with protein residues like hydrogen bonding, Pi electron density‐based interactions, hydrophobic, and non‐polar interactions as shown in docking Figures 2, 3, 4, 5, 6, 7.

**FIGURE 2:**
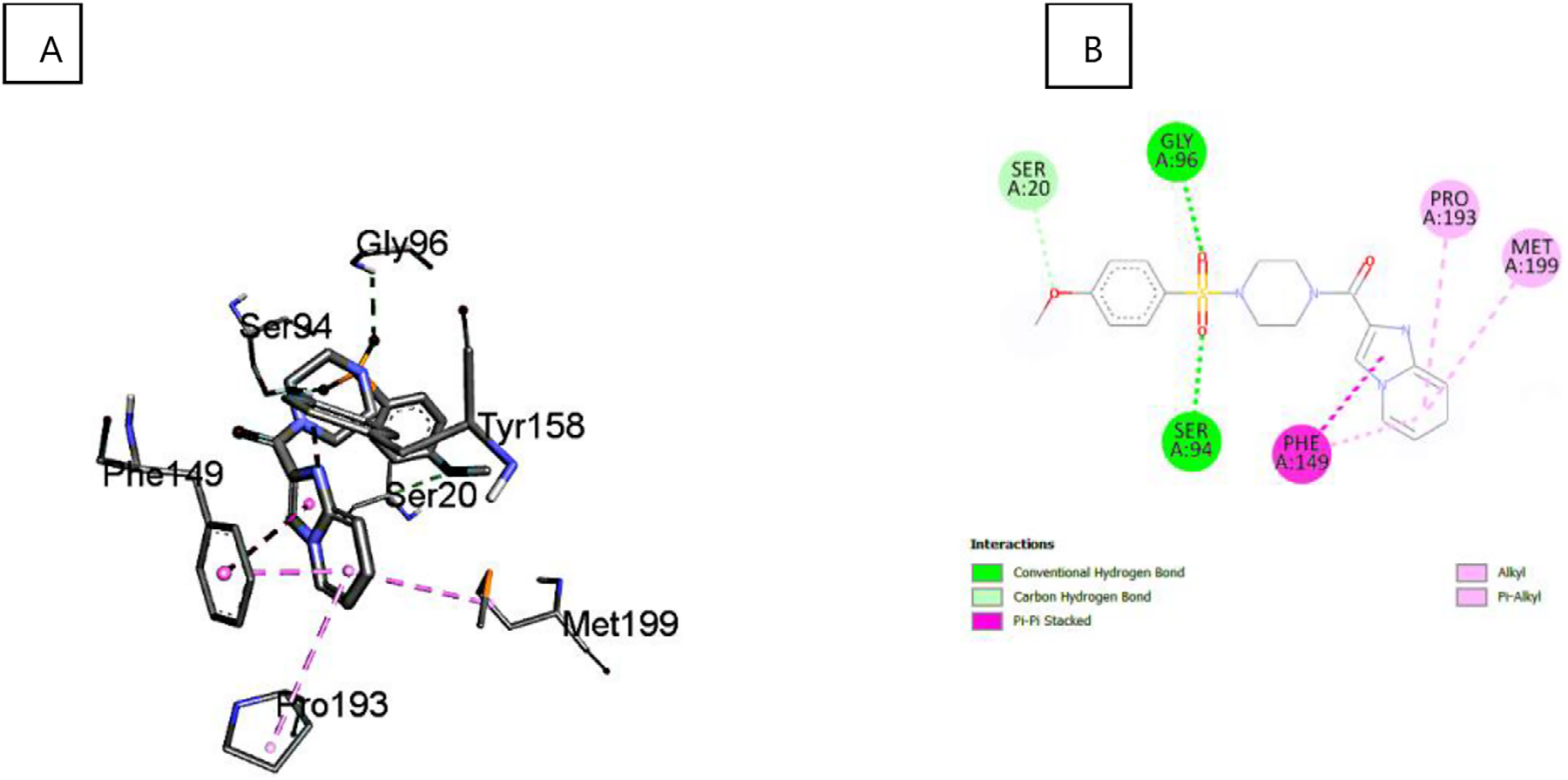
(A) 2D view of ligand **4a** in the bonding pocket of *PDB: 4TZK* protein. (B) The 3D view of the ligand **4a** in the active sites of the *PDB: 4TZK* protein.

**FIGURE 3:**
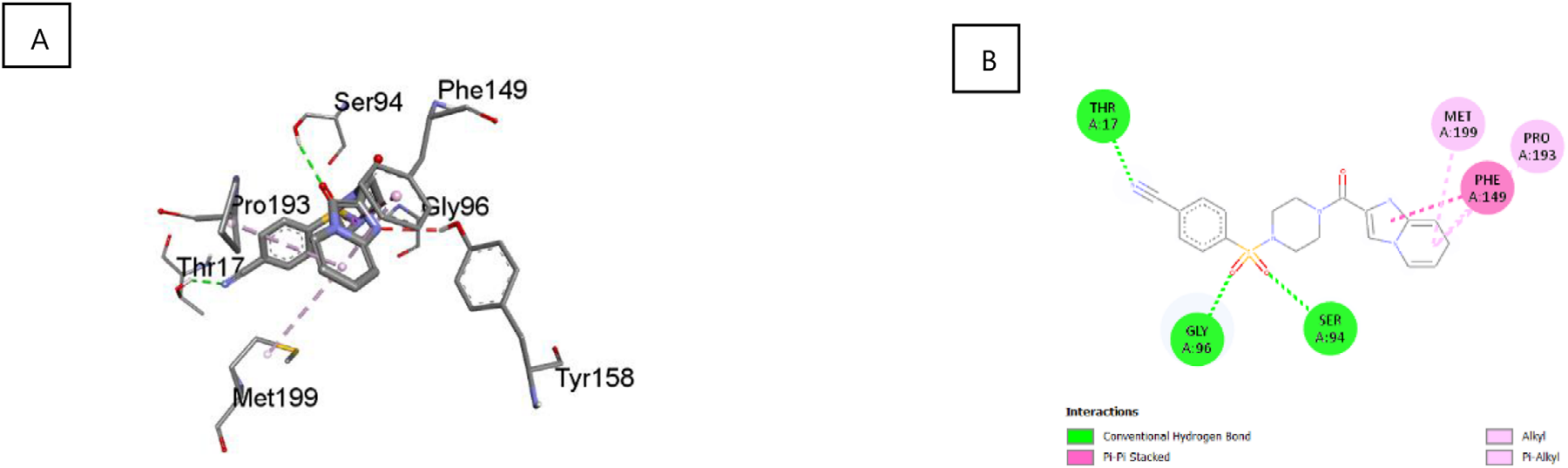
(A) 2D view of ligand **4b** in the bonding pocket of *PDB: 4TZK* protein. (B) The 3D view of the ligand **4b** in the active sites of the *PDB: 4TZK* protein.

**FIGURE 4:**
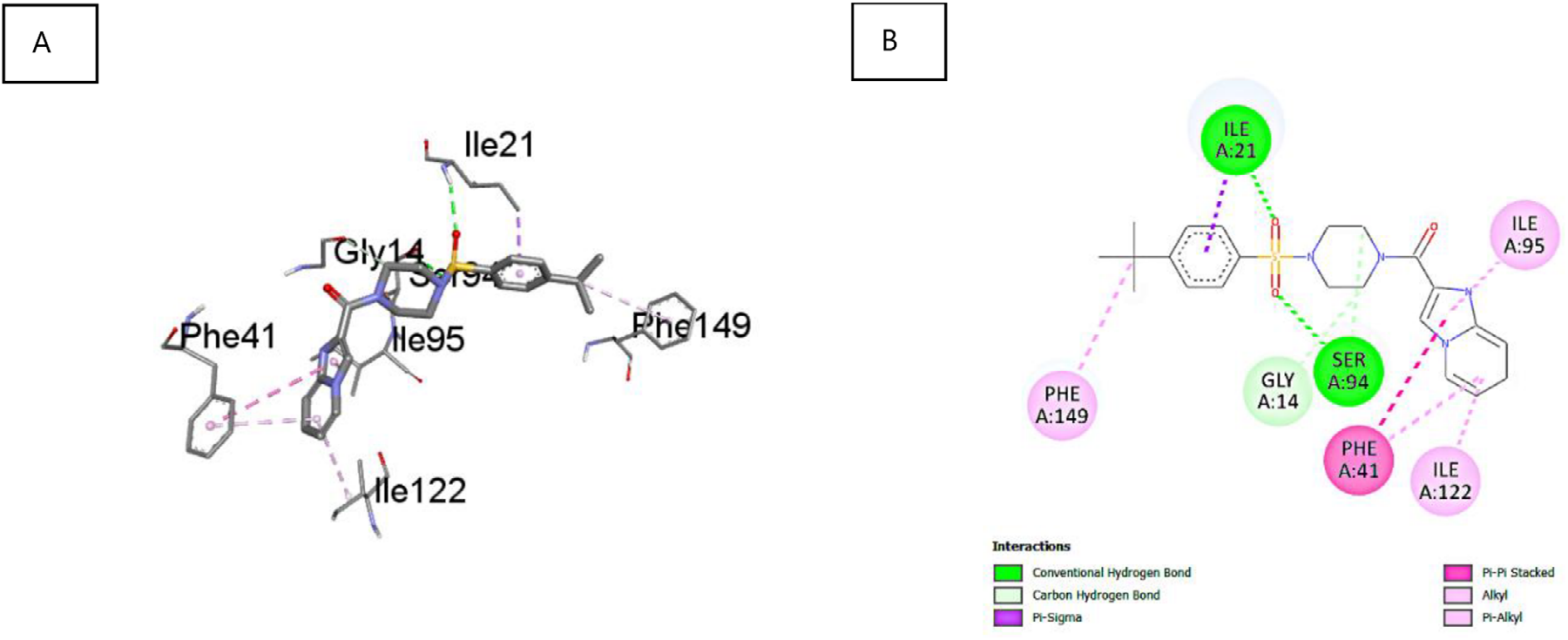
(A) 3D view of ligand **4d** in the bonding pocket of *PDB: 4TZK* protein. (B) The 2D view of the ligand **4d** in the active sites of the *PDB: 4TZK* protein.

**FIGURE 5:**
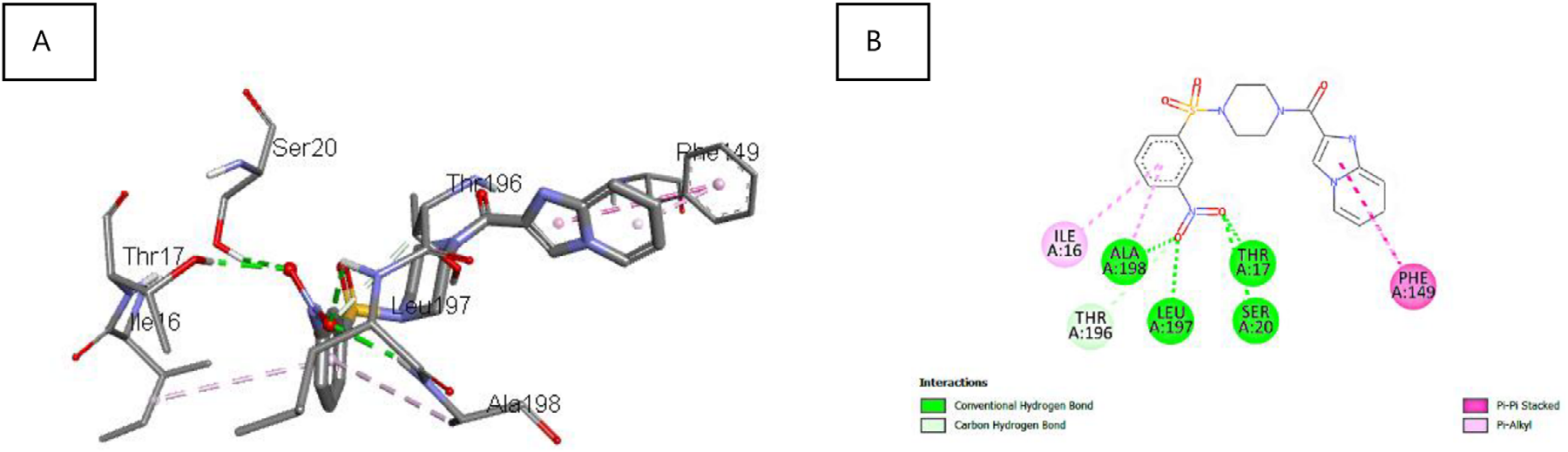
(A) 3D view of ligand **4e** in the bonding pocket of *PDB: 4TZK* protein. (B) The 2D view of the ligand **4e** in the active sites of the *PDB: 4TZK* protein.

**FIGURE 6:**
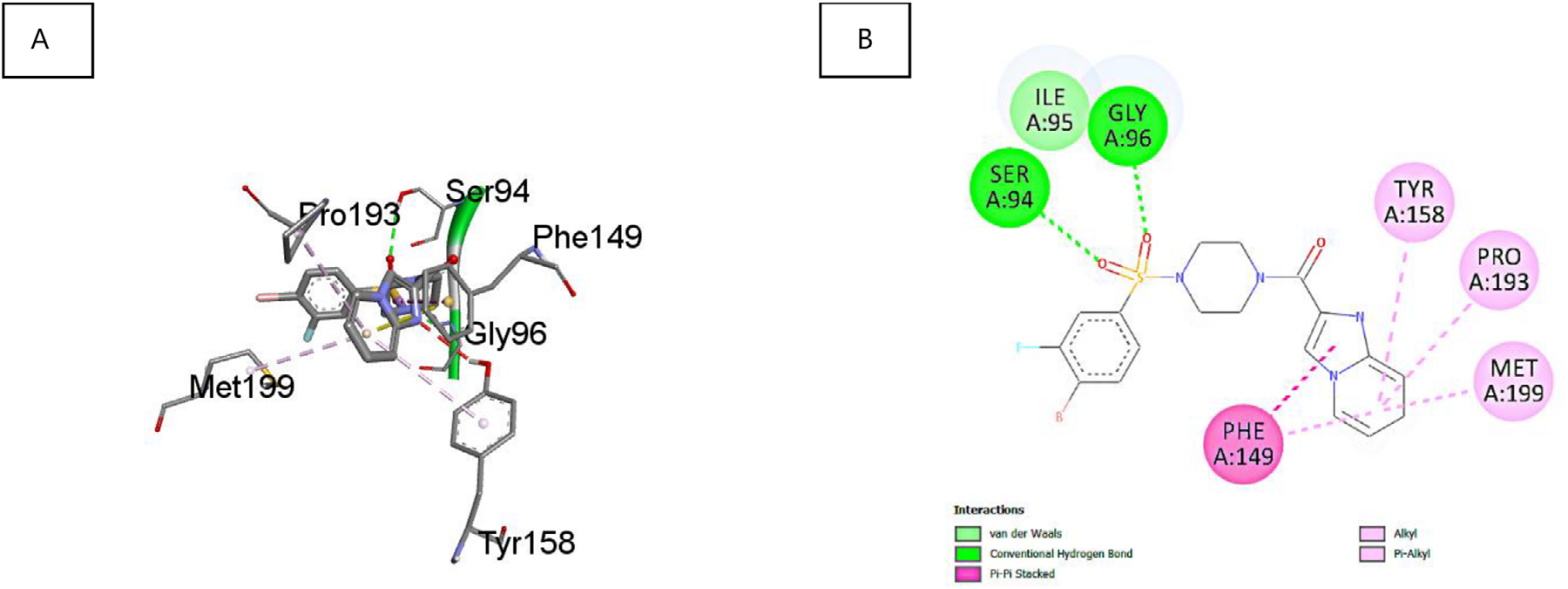
(A) 3D view of ligand **4g** in the bonding pocket of *PDB: 4TZK* protein. (B) The 2D view of the ligand **4g** in the active sites of the *PDB: 4TZK* protein.

**FIGURE 7:**
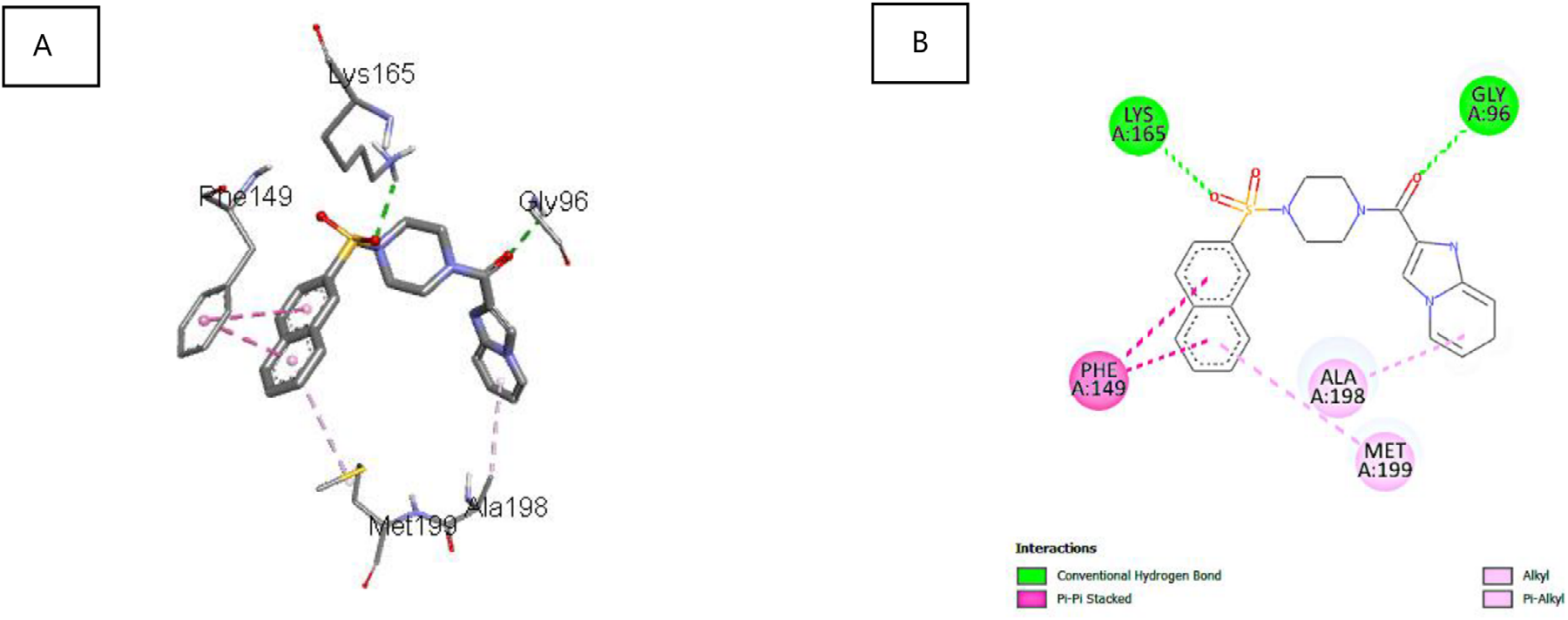
(A) 3D view of ligand **4k** in the bonding pocket of *PDB: 4TZK* protein. (B) The 2D view of the ligand **4k** in the active sites of the *PDB: 4TZK* protein.

##### 2.5.2.1 Molecular Docking Analysis of Compound 4a

The molecular docking analysis of compound **4a** with the *PDB: 4TZK* protein revealed that the ligand fits well within the active site of the enzyme (Figure 2A,B). The docking results show that compound **4a** forms conventional hydrogen bond interactions with Gly96 and Ser94, which help stabilize the ligand within the binding pocket.

Additionally, the aromatic moiety of the ligand exhibits π–π stacking interaction with Phe149, while π–alkyl interactions with Pro193 and Met199 contribute to hydrophobic stabilization of the ligand–protein complex. These interactions indicate that compound **4a** is favorably accommodated within the catalytic pocket of the 4TZK protein, supporting its potential biological activity.

##### 2.5.2.2 Molecular Docking Analysis of Compound 4b

The molecular docking study of compound **4b** with the PDB: 4TZK protein demonstrated that the ligand is well accommodated within the active site of the enzyme (Figure 3A,B). Compound **4b** forms conventional hydrogen bond interactions with Thr17, Gly96, and Ser94, which help stabilize the ligand within the binding pocket.

Furthermore, the aromatic ring of the ligand participates in π–π stacking interaction with Phe149, while π–alkyl interactions with Pro193 and Met199 contribute to hydrophobic stabilization of the ligand–protein complex. These interactions suggest that compound **4b** binds effectively within the catalytic site of the 4TZK protein, supporting its potential biological activity.

##### 2.5.2.3 Molecular Docking Analysis of Compound 4d

The molecular docking study of compound **4d** with the PDB: 4TZK protein revealed that the ligand fits well within the active site of the enzyme (Figure 4A,B). The docking results indicate that compound **4d** forms conventional hydrogen bond interactions with Ile21 and Ser94, while a carbon–hydrogen interaction with Gly14 further stabilizes the ligand within the binding pocket.

In addition, the aromatic ring of the ligand participates in π–π stacking interactions with Phe41, and π–alkyl/alkyl interactions with Ile95, Ile122, and Phe149, contributing to hydrophobic stabilization of the ligand–protein complex. These interactions suggest that compound **4d** is effectively accommodated within the catalytic site of the 4TZK protein, supporting its potential biological activity.

##### 2.5.2.4 Molecular Docking Analysis of Compound 4e

The docking study of compound **4e** with the *PDB: 4TZK* protein revealed that the ligand binds effectively within the active site of the enzyme (Figure 5A,B). Compound **4e** forms conventional hydrogen bond interactions with Thr17 and Ser20, which help stabilize the ligand within the binding pocket. Additionally, carbon–hydrogen interactions with Thr196 and Ala198 further contribute to the stability of the ligand–protein complex.

Moreover, the aromatic ring of the ligand exhibits π–π stacking interaction with Phe149, along with π–alkyl interactions with Ile16 and Leu197, which enhance hydrophobic stabilization. These interactions indicate that compound **4e** is well accommodated in the catalytic site of the 4TZK protein, supporting its potential biological activity.

##### 2.5.2.5 Molecular Docking Analysis of Compound 4g

The molecular docking study of compound **4g** with the *PDB: 4TZK* protein showed that the ligand is well accommodated within the active binding pocket (Figure 6A,B). Compound **4g** forms conventional hydrogen bond interactions with Gly96 and Ser94, which contribute to stabilizing the ligand within the active site.

Additionally, the aromatic moiety of the ligand participates in π–π stacking interaction with Phe149, while π–alkyl interactions with Tyr158, Pro193, and Met199 provide further hydrophobic stabilization of the ligand–protein complex. These interactions indicate that compound **4g** fits favorably within the catalytic site of the 4TZK protein, supporting its potential biological activity.

##### 2.5.2.6 Molecular Docking Analysis of Compound 4k

The molecular docking analysis of compound **4k** with the *PDB: 4TZK* protein showed that the ligand is well accommodated within the active binding pocket (Figure 7A,B). Compound **4k** forms conventional hydrogen bond interactions with Lys165 and Gly96, which help stabilize the ligand within the catalytic site.

Additionally, the aromatic rings of the ligand participate in π–π stacking interaction with Phe149, while π–alkyl interactions with Ala198 and Met199 further contribute to hydrophobic stabilization of the ligand–protein complex. These interactions suggest that compound **4k** binds effectively within the active site of the 4TZK protein, supporting its potential biological activity.

### 2.6 ADME Study

Early evaluation of pharmacokinetic properties is an important component of lead identification and optimization. Therefore, the synthesized imidazo[1,2‐*a*]pyridine derivatives (**4a–4k**) were subjected to computational ADMET analysis using the SwissADME platform, and the predicted parameters are summarized in Table 5.

**TABLE 5:** ADMET analysis of compounds (**4a–4k**).

Comp/property	TPSA	Ali Log S	GI absorption	Lipinski #violations	Ghose #violations	Veber #violations	Egan #violations	Muegge #violations
**4a**	92.6	−3.72	High	0	0	0	0	0
**4b**	107.16	−3.76	High	0	0	0	0	0
**4c**	83.37	−4.48	High	0	0	0	0	0
**4d**	83.37	−5.29	High	0	0	0	0	0
**4e**	129.19	−4.34	High	0	0	0	0	0
**4f**	92.6	−4.44	High	0	0	0	0	0
**4g**	83.37	−4.37	High	0	0	0	0	0
**4h**	92.6	−4.44	High	0	0	0	0	0
**4i**	83.37	−5.52	High	0	0	0	0	0
**4j**	83.37	−3.49	High	0	0	0	0	0
**4k**	83.37	−4.85	High	0	0	0	0	0

The calculated topological polar surface area (TPSA) values ranged from 83.37 Å^2^ to 129.19 Å^2^, indicating that the synthesized compounds possess a balanced polarity profile compatible with oral drug candidates. Compound **4e** exhibited the highest TPSA value (129.19 Å^2^), whereas compounds **4c**, **4d**, **4g**, **4i**, **4j**, and **4k** displayed lower TPSA values (83.37 Å^2^), which may favor membrane permeation while maintaining adequate aqueous compatibility.

Predicted aqueous solubility values (Ali Log S) varied between –3.49 and –5.52, suggesting moderate solubility across the series. Such values are generally considered acceptable for small‐molecule drug candidates and indicate that the introduction of different sulfonyl substituents did not adversely affect the overall solubility profile of the compounds.

A notable observation from the ADMET assessment was the uniformly high predicted gastrointestinal absorption for all synthesized derivatives. This finding suggests that the compounds possess physicochemical characteristics supportive of oral administration and may exhibit favorable absorption behavior following oral dosing.

Drug‐likeness evaluation using Lipinski, Ghose, Veber, Egan, and Muegge filters revealed no violations for any of the synthesized molecules. Compliance with these widely accepted criteria indicates that the designed derivatives fall within the physicochemical space commonly associated with orally active drugs and possess structural features favorable for further pharmaceutical development.

This computational analysis demonstrated that the synthesized imidazo[1,2‐*a*]pyridine derivatives combine promising biological activity with favorable predicted pharmacokinetic characteristics. The absence of drug‐likeness violations, together with acceptable polarity, moderate solubility, and high gastrointestinal absorption, supports the continued investigation of these compounds as potential antitubercular lead candidates.

## 3 Conclusions

A series of 11 novel imidazo[1,2‐*a*]pyridine‐based sulfonamide derivatives (**4a–4k**) was successfully synthesized and structurally characterized using spectroscopic and mass spectrometric techniques. Biological evaluation demonstrated moderate antitubercular activity among the synthesized compounds against *M. tuberculosis* H37Ra, with compounds **4b**, **4d**, and **4g** showing the highest activity within the series and warranting further structural optimization and biological investigation. The observed activity highlights the potential of the imidazo[1,2‐*a*]pyridine–piperazine–sulfonamide framework as a valuable scaffold for the development of new antimycobacterial agents.

The synthesized derivatives also exhibited moderate to good antioxidant activity in free‐radical scavenging assays and demonstrated minimal hemolytic effects against human erythrocytes, indicating a favorable preliminary safety profile. Molecular docking studies supported the experimental findings by revealing favorable interactions of the active compounds within the binding site of the selected tuberculosis‐associated target protein. Furthermore, in silico ADMET analysis predicted acceptable physicochemical and pharmacokinetic properties, including high gastrointestinal absorption and compliance with established drug‐likeness criteria.

The combined synthetic, biological, computational, and safety data identify compounds **4b**, **4d**, and **4g** as potential lead structures requiring further optimization and detailed mechanistic investigations aimed at the development of novel antitubercular therapeutics.

## 4 Experimental Section

All reagents and solvents were commercially available in analytical grade. Melting points were determined in capillary tube using a Veego melting point apparatus. Unless otherwise specified, yields refer to chromatographically and spectroscopy (^1^H NMR) homogeneous materials. The solvents used were filtered before use and dried using standard techniques. High‐resolution mass spectrometric analysis was performed for the compounds for which experimental HRMS data were available with a FTICR‐MS (Ionspec 7.0 T) spectrometer, Punjab University. Fourier‐transform infrared spectroscopy (FT‐IR) (KBr disc) (cm^−1^) were recorded at the Laboratory, Punjab University Chandigarh. The ^1^H NMR and ^13^C NMR spectra were recorded in CDCl_3_ solutions on a Bruker a Vance Neo 400 and 100 MHz NMR Spectrometer (Saif, Punjab University) spectrometer. Chemical shifts are reported in parts per million (ppm) relative to CDCl_3_ (7.27 ppm) for ^1^H‐NMR data and CDCl_3_ (77.0 ppm) for ^13^C‐NMR data. The following abbreviations were used to explain multiplicities: s = singlet, d = doublet, t = triplet, q = quartet, m = multiplet, dt = doublet of triplet, and bs = broad singlet.

### 4.1 Synthesis of Imidazo[1,2‐*a*]pyridin‐2‐yl(piperazin‐1‐yl)methanone Hydrochloride **(2)** [37]

A mixture of *tert*‐butyl 4‐(imidazo[1,2‐*a*]pyridine‐2‐carbonyl)piperazine‐1‐carboxylate (**1**) (10 mmols) and HCl (40 mmols) was stirred in 10 mL 1,4‐dioxane at room temperature. The progress of the reaction was monitored by TLC and after completion of reaction, reaction mass was concentrated under vacuum to dryness to afford off white solid. The obtained crude material was triturated with hexane (25.00 mL) to afford final product (mobile phase: CHCl_3_:CH_3_OH = 9:1).

### 4.2 Synthesis of Imidazopyridine Derivatives (**4a–4k**)

A mixture of imidazo[1,2‐*a*]pyridin‐2‐yl(piperazin‐1‐yl)methanone hydrochloride (**2**) (10 mmols) and DIPEA (30 mmols) in DMF (6 mL) was stirred for 5 min at 0°C followed by addition of sulfonyl chloride derivatives (**3a–3k**) (11 mmols). Then reaction mixture was stirred at 60°C for 8 h. Progress of the reaction was monitored by TLC (5% methanol in DCM, UV, iodine, and ninhydrin). After completion of reaction, the reaction mass was poured in ice cold water and extracted with ethyl acetate. The organic layer was dried over sodium sulphate and concentrated under vacuum. The obtained crude material was purified by column chromatography through silica gel to afford pure product.

The FT‐IR, ^1^H NMR, ^13^C NMR, and mass spectra of synthesized compounds are included in Figures S1–S43.

#### 4.2.1 Imidazo[1,2‐a]pyridin‐2‐yl(4‐((4‐methoxyphenyl)sulfonyl)piperazin‐1‐yl)methanone **(4a)**

Off white solid; yield 65%–66%; **m.p**. 192.7°C; **IR (in cm^−1^)**: 3424.35, 1612.75, 1524.70, 1329.89, 1255.36, 1031.58, 755.22; **^1^H NMR (400 MHz, CDCl_3_)**: δ 8.09 (d, *J* = 6.8 Hz, 1H), 8.02 (s, 1H), 7.64 (d, *J* = 8.9 Hz, 2H), 7.51 (d, *J* = 9.2 Hz, 1H), 7.19 (dd, *J* = 12.0, 3.9 Hz, 1H), 6.95 (d, *J* = 8.9 Hz, 2H), 6.81 (t, *J* = 6.5 Hz, 1H), 4.72 (bs, 2H), 4.40 (bs, 2H), 3.83 (s, 3H), 3.09–3.03 (t, 4H); **^13^C NMR (100 MHz, CDCl_3_)**: δ 163.23, 152.67, 135.25, 134.97, 129.87, 126.69, 126.21, 126.10, 125.94, 125.86, 118.31, 116.96, 114.38, 113.53, 55.64, 46.66, 46.02; **elemental analysis (%)**: C, 56.99; H, 5.03; N, 13.99; O, 15.98; S, 8.01; **molecular formula**: C_19_H_20_N_4_O_4_S; **exact mass [M]^+^**: 400.1205, **found [M + 1]^+^**: 401.1271.

#### 4.2.2 4‐((4‐(Imidazo[1,2‐a]pyridine‐2‐carbonyl)piperazin‐1‐yl)sulfonyl)benzonitrile **(4b)**

Off white solid; yield 65%–66%; **m.p**. 198.6°C; **IR (in cm^−1^)**: 3414.05, 1610.10, 1524.87, 1340.72, 1253.94, 1027.23, 749.89; **^1^H NMR (400 MHz, CDCl_3_)**: δ 8.12 (dt, *J* = 6.9, 1.1 Hz, 1H), 8.08 (d, *J* = 0.5 Hz, 1H), 7.93–7.82 (m, 4H), 7.55 (dd, *J* = 9.2, 0.8 Hz, 1H), 7.25–7.21 (m, 1H), 6.86 (td, *J* = 6.8, 1.1 Hz, 1H), 4.53 (bs, 2H), 3.91 (bs, 2H), 3.28–3.09 (t, 4H); **^13^C NMR (100 MHz, CDCl_3_)**: δ 163.09, 143.93, 141.66, 139.85, 133.02, 128.28, 126.13, 125.99, 121.34, 121.03, 118.21, 117.09, 116.77, 113.59, 45.82, 42.87, 42.26; **elemental analysis (%)**: C, 57.71; H, 4.33; N, 17.71; O, 12.14; S, 8.11; **molecular formula**: C_19_H_17_N_5_O_3_S; **exact mass [M]^+^**: 395.1052, **found [M + 1]^+^**: 396.1123.

#### 4.2.3 Imidazo[1,2‐a]pyridin‐2‐yl(4‐((4‐(trifluoromethyl)phenyl)sulfonyl)piperazin‐1‐yl)methanone**(4c)**

White solid; yield 60%–62%; **m.p**. 202.1°C, **IR (in cm^−1^)**: 3279.09, 1619.72, 1524.84, 1325.47, 1255.59, 1015.54, 759.56; **^1^H NMR (400 MHz, CDCl_3_)**: δ 8.09 (d, *J* = 6.8 Hz, 1H), 8.04 (s, 1H), 7.87 (d, *J* = 8.3 Hz, 2H), 7.79 (t, *J* = 7.7 Hz, 2H), 7.51 (d, *J* = 9.2 Hz, 1H), 7.24–7.14 (m, 1H), 6.82 (t, *J* = 6.5 Hz, 1H), 4.68 (bs, 2H), 3.86 (bs, 2H), 3.20–3.09 (t, 4H); **^13^C NMR (100 MHz, CDCl_3_)**: δ 163.42, 143.92, 140.17, 139.05, 135.58, 134.95, 134.03, 128.23, 126.42, 126.09, 125.97, 118.27, 117.09,113.60, 103.86, 46.05, 44.89; **elemental analysis (%)**: C, 52.05; H, 3.91; F, 13.00; N, 12.78; O, 10.95; S, 7.31; **molecular formula**: C_19_H_17_F_3_N_4_O_3_S; **exact mass [M]^+^**: 438.0973, **found [M + 1]^+^**: 439.1048.

#### 4.2.4 (4‐((4‐(tert‐Butyl)phenyl)sulfonyl)piperazin‐1‐yl)(imidazo[1,2‐a]pyridin‐2‐yl)methanone **(4d)**

Off white solid; yield 68%–70%; **m.p**. 205.01°C; **IR (in cm^−1^)**: 3442.34, 1669.05, 1620.29, 1524.66, 1328.24, 1251.34, 1054.46, 744.56; **^1^H NMR (400 MHz, CDCl_3_)**: δ 8.11 (dt, *J* = 6.9, 1.1 Hz, 1H), 8.06 (d, *J* = 0.5 Hz, 1H), 7.72–7.61 (m, 2H), 7.60–7.48 (m, 4H), 6.85 (td, *J* = 6.8, 1.1 Hz, 1H), 4.47 (bs, 2H), 3.89 (bs, 2H), 3.17–3.11 (m, 4H), 1.35 (s, 9H); **^13^C NMR (100 MHz, CDCl_3_)**: δ 163.21, 156.88, 143.97, 140.16, 132.26, 132.14, 127.67, 126.94, 126.23, 126.07, 126.02, 125.90, 118.31, 116.92, 113.56, 55.08, 43.18, 35.04, 30.97; **elemental analysis (%)**: C, 61.95; H, 6.14; N, 13.14; O, 11.25; S, 7.52; **molecular formula**: C_22_H_26_N_4_O_3_S; **exact mass** [M]^+^: 426.1726.

#### 4.2.5 Imidazo[1,2‐a]pyridin‐2‐yl(4‐((3‐nitrophenyl)sulfonyl)piperazin‐1‐yl)methanone **(4e)**

Light yellow solid; yield 80%–85%; **m.p**. 188.7°C; **IR (in cm^−1^)**: 3432.28, 1609.29, 1540.36, 1332.02, 1256.20, 1069.50, 748.88; **^1^H NMR (400 MHz, CDCl_3_)**: δ 8.56 (dd, *J* = 4.6, 2.8 Hz, 1H), 8.49–8.39 (m, 1H), 8.14–8.01 (m, 2H), 7.77 (q, *J* = 7.9 Hz, 1H), 7.51 (d, *J* = 9.2 Hz, 1H), 7.27 (s, 1H), 7.21 (ddd, *J* = 9.1, 6.8, 1.1 Hz, 1H), 6.87–6.76 (m, 1H), 4.70 (bs, 4H), 3.25–3.12 (t, 4H); **^13^C NMR (100 MHz, CDCl_3_)**: δ 163.13, 159.06, 143.91, 139.73, 137.68, 133.15, 130.80, 127.56, 126.15, 126.00, 122.66, 120.14, 118.14, 117.01, 113.57, 113.43, 44.90, 43.19; **elemental analysis (%)**: C, 52.04; H, 4.12; N, 16.86; O, 19.26; S, 7.72; **molecular formula**: C_18_H_17_N_5_O_5_S; **exact mass [M]^+^**: 415.0950, **found [M + 1]^+^**: 416.1022.

#### 4.2.6 (4‐((4‐Bromo‐2‐methoxyphenyl)sulfonyl)piperazin‐1‐yl)(imidazo[1,2‐a]pyridin‐2‐yl)methanone **(4f)**

Off white solid; yield 75%–80%; **m.p**. 200.8°C; **IR (in cm^−1^)**: 3424.00, 1632.87, 1525.51, 1350.77, 1255.82, 1039.72, 764.08; **^1^H NMR (400 MHz, CDCl_3_)**: δ 8.11 (d, *J* = 6.8 Hz, 1H), 8.06 (s, 1H), 7.97 (d, *J* = 2.5 Hz, 1H), 7.59 (dd, *J* = 8.8, 2.5 Hz, 1H), 7.53 (d, *J* = 9.2 Hz, 1H), 7.21 (dd, *J* = 9.1, 6.8 Hz, 1H), 6.88 (d, *J* = 8.8 Hz, 1H), 6.83 (s, 1H), 4.37 (bs, 2H), 3.85 (d, m, 6H), 3.29 (s, 3H); **^13^C NMR (100 MHz, CDCl_3_)**: δ 187.52, 186.93, 186.09, 174.42, 173.90, 170.36, 161.41, 160.49, 152.04, 137.83, 136.40, 135.26, 133.77, 114.34, 56.51, 46.05, 44.31; **elemental analysis (%)**: C, 47.61; H, 4.00; Br, 16.67; N, 11.69; O, 13.35; S, 6.69; **molecular formula**: C_19_H_19_BrN_4_O_4_S; **exact mass [M]^+^**: 478.0310, **found [M + 1]^+^**: 479.0389.

#### 4.2.7 (4‐((4‐Bromo‐3‐fluorophenyl)sulfonyl)piperazin‐1‐yl)(imidazo[1,2‐a]pyridin‐2‐yl)methanone **(4g)**

Off white solid; yield 65%–70%; **m.p**. 201.5°C; **IR (in cm^−1^)**: 3432.18, 1609.29, 1540.36, 1332.02, 1256.20, 1069.50, 748.88; **^1^H NMR (400 MHz, CDCl_3_)**: δ 8.10 (d, *J* = 6.8 Hz, 1H), 8.04 (s, 1H), 7.72 (dd, *J* = 8.3, 6.5 Hz, 1H), 7.56–7.43 (m, 2H), 7.39 (dd, *J* = 8.3, 1.5 Hz, 1H), 7.21 (dd, *J* = 9.1, 1.1 Hz, 1H), 6.86–6.76 (m, 1H), 4.46 (s, 2H), 3.86 (s, 2H), 3.15–3.09 (m, 4H); **^13^C NMR (100 MHz, CDCl_3_)**: δ 163.12, 157.91, 143.90, 139.94, 136.67, 134.66, 126.09, 125.95, 124.28, 118.29, 117.09, 115.97, 115.23, 113.59, 46.03, 38.77; **elemental analysis (%)**: C, 46.26; H, 3.45; Br, 17.10; F, 4.07; N, 11.99; O, 10.27; S, 6.86; **molecular formula**: C_18_H_16_BrFN_4_O_3_S; **exact mass [M]^+^**: 466.0111, **found [M+1]^+^**: 467.0184.

#### 4.2.8 (4‐((5‐Bromo‐2‐methoxyphenyl)sulfonyl)piperazin‐1‐yl)(imidazo[1,2‐a]pyridin‐2‐yl)methanone **(4h)**

Off white solid; yield 75%–80%; **m.p**. 198.4°C; **IR (in cm^−1^)**: 3418.38, 1618.81, 1526.28, 1345.03, 1283.88, 1038.84, 756.28; **^1^H NMR (400 MHz, CDCl_3_)**: δ 8.13 (d, *J* = 6.8 Hz, 1H), 8.09 (s, 1H), 7.62 (dd, *J* = 8.8, 2.5 Hz, 1H), 7.57 (d, *J* = 9.2 Hz, 1H), 7.29–7.21 (m, 2H), 6.94–6.83 (m, 2H), 4.42 (s, 2H), 3.91 (s, 3H), 3.87 (s, 2H), 3.38 (d, *J* = 31.3 Hz, 4H); **^13^C NMR (100 MHz, CDCl_3_)**: δ 163.28, 160.20, 155.96, 153.58, 143.95, 140.13, 137.31, 133.94, 127.70, 126.11, 125.89, 118.25, 116.84, 114.18, 113.53, 112.41, 110.27, 56.38, 44.87; **elemental analysis (%)**: C, 47.61; H, 4.00; Br, 16.67; N, 11.69; O, 13.35; S, 6.69; **molecular formula**: C_19_H_19_BrN_4_O_4_S; **exact mass [M]^+^**: 478.0310, **found [M+1]^+^**: 479.0385

#### 4.2.9 Imidazo[1,2‐a]pyridin‐2‐yl(4‐((2,4,5‐trichlorophenyl)sulfonyl)piperazin‐1‐yl)methanone **(4i)**

Off white solid; yield 75%–80%; **m.p**. 182.4°C; **IR (in cm^−1^)**: 3418.02, 1608.54, 1540.42, 1351.59, 1256.39, 1069.86, 748.81; **^1^H NMR (400 MHz, CDCl_3_)**: δ 8.22–8.07 (m, 3H), 7.65 (s, 1H), 7.62–7.50 (m, 2H), 6.87 (t, *J* = 6.5 Hz, 1H), 4.48 (s, 2H), 3.88 (s, 2H), 3.47–3.40 (t, 4H); **^13^C NMR (100 MHz, CDCl3)**: δ 163.13, 152.65, 147.77, 143.70, 140.46, 139.86, 138.42, 135.79, 133.43, 133.22, 133.10, 132.04, 125.91, 118.38, 116.67, 113.44, 55.05, 43.16; **elemental analysis (%)**: C, 45.64; H, 3.19; Cl, 22.45; N, 11.83; O, 10.13; S, 6.77; **molecular formula**: C_18_H_15_Cl_3_N_4_O_3_S; **exact mass [M]^+^**: 471.9930, **found [M + 1]^+^**: 473.003.

#### 4.2.10 (4‐(Benzylsulfonyl)piperazin‐1‐yl)(imidazo[1,2‐a]pyridin‐2‐yl)methanone **(4j)**

Off white solid; yield 80%–85%; **m.p**. 189.1°C; **IR (in cm^−1^)**: 3427.21, 1611.34, 1539.60, 1384.13, 1258.53, 1033.47, 753.79; **^1^H NMR (400 MHz, CDCl_3_)**: δ 8.11 (dt, *J* = 6.8, 1.1 Hz, 1H), 8.06 (d, *J* = 0.5 Hz, 1H), 7.52 (dd, *J* = 9.2, 0.8 Hz, 1H), 7.33 (dd, *J* = 5.1, 1.8 Hz, 5H), 7.20 (ddd, *J* = 9.2, 6.7, 1.2 Hz, 1H), 6.82 (td, *J* = 6.8, 1.1 Hz, 1H), 4.28 (s, 2H), 4.21 (m, *J* = 5.8 Hz, 2H), 3.70 (bs, 2H), 3.19 (m, 4H); **^13^C NMR (100 MHz, CDCl_3_)**: δ 163.21, 143.93, 140.10, 139.34, 130.63, 128.84, 128.40, 127.45, 126.12, 125.87, 118.24, 116.90, 116.65, 113.52, 57.10, 43.17, 38.82.; **elemental analysis (%)**: C, 59.36; H, 5.24; N, 14.57; O, 12.48; S, 8.34; **molecular formula**: C_19_H_20_N_4_O_3_S; **exact mass [M]^+^**: 384.1256, **found [M + 1]^+^**: 385.1325.

#### 4.2.11 Imidazo[1,2‐a]pyridin‐2‐yl(4‐(naphthalen‐2‐ylsulfonyl)piperazin‐1‐yl)methanone **(4k)**

Off white solid; yield 60%–65%; m.p. 188.2°C; **IR (in cm^−1^)**: 3457.03, 1613.10, 1552.38, 1324.74, 1251.16, 1051.15, 746.97; **^1^H NMR (400 MHz, CDCl_3_)**: δ 8.30 (s, 1H), 8.06 (d, *J* = 6.7 Hz, 1H), 7.97 (dd, *J* = 16.6, 8.0 Hz, 3H), 7.92–7.87 (m, 1H), 7.71 (dd, *J* = 8.6, 1.7 Hz, 1H), 7.67–7.56 (m, 2H), 7.50 (d, *J* = 9.1 Hz, 1H), 7.22–7.15 (m, 1H), 6.80 (t, *J* = 6.8 Hz, 1H), 4.45 (s, 2H), 3.86 (s, 2H), 3.17 (s, 4H); **^13^C NMR (100 MHz, CDCl_3_)**: δ 163.08, 162.83, 143.87, 139.96, 138.74, 134.93, 132.30, 132.13, 129.42, 129.17, 129.09, 129.01, 127.91, 127.69, 126.07, 125.85, 122.74, 118.20, 116.94, 113.49, 46.34, 44.90; **elemental analysis (%)**: C, 62.84; H, 4.79; N, 13.32; O, 11.41; S, 7.62; **molecular formula**: C_22_H_20_N_4_O_3_S; **exact mass [M]^+^**: 420.1256, **found [M + 1]^+^**: 421.1319.

### 4.3 Antituberculosis Activity

The minimum inhibitory concentration (MIC) of the synthesized compounds **4a–4k** against *M. tuberculosis* H37Ra was determined using the microplate Alamar Blue assay (MABA) [38]. *Mycobacterium tuberculosis* cultures were grown to the mid‐logarithmic phase until an optical density (OD_600_) of 0.6, corresponding to approximately 5 × 10^7^ CFU/mL, was reached. The bacterial suspension was appropriately diluted, and 100 µL (≈ 5 × 10^4^ CFU) was dispensed into each well of sterile 96‐well microplates.

Test compounds **4a–4k** were prepared as serial two‐fold dilutions over the desired concentration range and added to the wells in triplicate. DMSO served as the negative control, while rifampicin was included as the positive control. Each experiment was performed in three independent biological replicates. The plates were sealed and incubated at 37°C for 6 days under static conditions.

After incubation, 10% (v/v) Alamar Blue reagent was added to each well, and the plates were further incubated to allow color development. Bacterial growth was assessed both visually and spectrophotometrically. A blue‐to‐pink color change indicated bacterial growth, whereas retention of the blue color indicated growth inhibition. Absorbance was recorded at 600 nm using a microplate reader.

The MIC was defined as the lowest concentration of the test compounds **4a–4k** that completely inhibited visible mycobacterial growth, as evidenced by the absence of color change. Absorbance values were expressed as mean ± standard deviation (SD). Percentage growth inhibition was calculated relative to the growth control. Statistical analysis was performed using appropriate software and differences were considered significant at *p* < 0.05.

### 4.4 Antioxidant Activity

The antioxidant activity of tested compounds (**4a–4k**) evaluated by reported 2,2‐diphenyl‐1‐picrylhydrazyl radical scavenging assay (DPPH) and hydroxyl radical assay [39]. Procedure provided in Supporting Information.

### 4.5 Hemolytic Activity (Cytotoxicity)

The hemolytic activities using human RBCs for the compounds (**4a–4k**) were determined by using reported protocol [40]. Procedure provided in Supporting Information.

### 4.6 In Silico Study

#### 4.6.1 Target Prediction

Based on the observed antimycobacterial activity in the microplate Alamar Blue assay (MABA), molecular docking studies were performed to explore possible interactions of the synthesized compounds with the selected *M. tuberculosis* target, InhA (PDB ID: 4TZK).

#### 4.6.2 Molecular Docking Studies

Computer‐aided drug design (CADD) is a constructive approach aiding drug discovery, as it allows us to screen and analyze a vast number of compounds efficiently and effectively. In the present study, the molecular docking studies for compounds **4a–4k** were carried out. The studies were performed employing silicon docking software Autodock Vina to design and analyze a series of derivatives [41].

#### 4.6.3 Target Preparation

The three‐dimensional x‐ray crystal structure of *M. tuberculosis* enoyl‐[acyl‐carrier‐protein] reductase (InhA) in complex with 1‐cyclohexyl‐*N*‐(3,5‐dichlorophenyl)‐5‐oxopyrrolidine‐3‐carboxamide (PDB ID: 4TZK; resolution 1.62 Å) was obtained from the RCSB Protein Data Bank (https://www.rcsb.org/). The native ligands from proteins were deleted and proteins were prepared for molecular docking using PyMOL [42]. All the water molecules were removed using PyMOL and proteins were saved in the pdb format used for molecular docking.

#### 4.6.4 Ligand Preparation

All the ligands were carefully prepared for molecular docking using Chem 3D 20.0. and PyMOL to ensure precise and reliable results. The energy minimization was done with the Chem 3D 20.0 and all the MM2 molecules were saved in the pdb format used for molecular docking [43].

#### 4.6.5 System Validation and Molecular Docking

The validation of all the docking results was performed with the “Autodock Vina” docking protocol performed by re‐docking the native ligand of the selected protein and all the results were rechecked whether all the ligands showing similar interaction as per the native ligand were superimposable with each other [44]. The accuracy of the docking protocol was evaluated and identified the binding sites by comparing the algorithm's ability to replicate the positioning of the original co‐crystallized ligand. This involved calculating the root mean square deviation (RMSD) between the docked position and the native pose for each protein complex. After validating the docking protocol, the designed compounds were docked into the binding site of each prepared protein crystal structure using default parameters.

### 4.7 ADMET Study

In the final phase of the study, the compounds (**4a–4k**) identified from docking and binding energy evaluations were subjected to in silico ADMET analysis. The pharmacokinetic and drug‐likeness profiles were predicted using the SwissADME web server [45]. This platform was selected owing to its several advantages in early‐stage drug discovery.

SwissADME provides a robust and comprehensive evaluation of absorption, distribution, metabolism, excretion, and toxicity (ADMET) related parameters, thereby enabling rapid identification of potential liabilities before experimental validation [46]. The tool integrates multiple computational models to predict lipophilicity, water solubility, gastrointestinal (GI) absorption, blood‐brain barrier (BBB) permeability, cytochrome P450 (CYP) interactions, and rule‐based filters for drug‐likeness.

## Author Contributions

**Hanmant N. Chaugule**: conceptualization, methodology. **Shravan Y. Jadhav**: conceptualization, supervision. **Sujay A. Ghorpade**: software. **Dayanand C. Kore**: data curation. **Vaibhav R. Mane**: investigation. **Umakant M. Kadampalle**: validation. **Vijay H. Masand**: visualization. **Magdi E. A. Zaki**: funding acquisition. **Rohit S. Bavi**: project administration. **Samin A. Shaikh**: writing – original draft.

## Conflicts of Interest

The authors declare no conflicts of interest.

## Declaration of Generative AI and AI‐Assisted Technologies in the Writing Process

During the preparation and revision of this work, the authors used ChatGPT in order to improve the readability of the content. After using this tool, the authors reviewed and edited the content as needed and took full responsibility for the content of the publication.

## Supporting information



The Supporting Information accompanying this manuscript contains the FT‐IR, ^1^H NMR, and ^13^C NMR, spectra of the synthesized compounds (**4a**–**4k**), together with the available HRMS spectra of selected compounds (Figures S1–S43).
**Supporting File 1**: cbdv71854‐sup‐0001‐SuppMat.docx

## Data Availability

The data that supports the findings of this study are available in the Supporting Information of this article.

## References

[1] Z. Fatima , D. Purkait , S. Rehman , S. Rai , and S. Hameed , “Multidrug Resistance: A Threat to Antibiotic Era,” in Biological and Environmental Hazards, Risks, and Disasters (Elsevier, 2023), 197–220.

[2] V. Yazbeck , E. Alesi , J. Myers , M. H. Hackney , L. Cuttino , and D. A. Gewirtz , “An Overview of Chemotoxicity and Radiation Toxicity in Cancer Therapy,” Advances in Cancer Research 155 (2022): 1–27. 10.1016/bs.acr.2022.03.007 35779872

[3] L. A. Ferreira , L. Caruso , and N. F. Nadur , Imidazo[1,2‐a]pyridines in Medicinal Chemistry: Recent Advances in Synthesis and Biological Activities (ACS Omega, 2026). 10.1021/acsomega.5c08365 PMC12824820 41585699

[4] M. Koudad , S. Dadou , F. Abrigach , et al., “Synthesis and Antimicrobial, Antioxidant, ADME‐T, and Molecular Docking Studies of Imidazo[1,2‐*a*]pyridine Derivatives,” Russian Journal of Organic Chemistry 59, no. 7 (2023): 1237–1247.

[5] G. Jose , T. S. Kumara , G. Nagendrappa , et al., “Synthesis, Molecular Docking and Anti‐Mycobacterial Evaluation of New Imidazo[1,2‐*a*]pyridine‐2‐carboxamide Derivatives,” European Journal of Medicinal Chemistry 89 (2015): 616–627. 10.1016/j.ejmech.2014.10.079 25462270

[6] M. B. Kadam , S. A. Shaikh , S. S. Chobe , et al., “Triazolo‐Pyridine Derivatives: Synthesis, Characterization and Biological Evaluation,” Journal of Molecular Structure 1356 (2025): 144958.

[7] K. Armendariz‐Barrientos , L. A. Pérez , S. Lagunas‐Rivera , et al., “Novel *N*‐Quaternary Coumarin‐3‐yl‐imidazo[1,2‐*a*]pyridines as Fluorescent Hybrids: Their Synthesis and Biological Evaluation in Cancer Cells,” Results in Chemistry 13 (2025): 101959.

[8] M. Sharma and P. Prasher , “C2‐Functionalized Imidazo[1,2‐*a*]pyridine: Synthesis and Medicinal Relevance,” Synthetic Communications 52, no. 11‐12 (2022): 1337–1356.

[9] S. Y. Jadhav , S. P. Shirame , S. D. Kulkarni , S. B. Patil , S. K. Pasale , and R. B. Bhosale , “PEG Mediated Synthesis and Pharmacological Evaluation of Some Fluoro Substituted Pyrazoline Derivatives as Antiinflammatory and Analgesic Agents,” Bioorganic & Medicinal Chemistry Letters 23, no. 9 (2013): 2575–2578. 10.1016/j.bmcl.2013.02.105 23541672

[10] M. Moutaouakil , S. Moutaouakil , O. Roby , S. Tighadouini , and R. Saddik , “Synthesis and Evaluation of Promising Antibacterial, Antifungal, and Antioxidant Activities for New Imidazo[1,2‐a]pyridine‐Derived Imines,” ChemistrySelect 9, no. 21 (2024): e202401098.

[11] S. Y. Jadhav , R. B. Bhosale , S. P. Shirame , S. B. Patil , and S. D. Kulkarni , “PEG Mediated Synthesis and Biological Evaluation of Asymmetrical Pyrazole Curcumin Analogues as Potential Analgesic, Anti‐Inflammatory and Antioxidant Agents,” Chemical Biology & Drug Design 85, no. 3 (2015): 377–384. 10.1111/cbdd.12416 25130070

[12] N. A. Peerzade , S. Y. Jadhav , B. D. Varpe , A. A. Kulkarni , and R. B. Bhosale , “Green Synthesis, Molecular Docking, In Silico ADME and Biological Evaluation of Methoxy Substituted 1,5‐Benzodiazepines as Potential Antioxidant, Anti‐Inflammatory, and Antidiabetic Agents,” Polycyclic Aromatic Compounds 42, no. 7 (2022): 3939–3952.

[13] H. R. Suryavanshi , “Synthesis and Biological Activities of Piperazine Derivatives as Antimicrobial and Antifungal Agents,” Organic Communications 10, no. 3 (2017): 228.

[14] D. C. Batista , D. P. Silva , I. F. Florentino , et al., “Anti‐Inflammatory Effect of a New Piperazine Derivative:(4‐Methylpiperazin‐1‐yl)(1‐Phenyl‐1H‐pyrazol‐4‐yl) Methanone,” Inflammopharmacology 26, no. 1 (2018): 217–226. 10.1007/s10787-017-0390-8 28825161

[15] N. A. Al‐Masoudi , R. S. Jihad , N. A. Abdul‐Rida , et al., “Synthesis, Antiproliferative, Antioxidant Activities, In Silico Studies, DFT Analysis and Molecular Dynamics Simulation of Novel 1‐(4‐Chlorobenzhydryl) Piperazine Derivatives,” Journal of Molecular Structure 1298 (2024): 137028.

[16] S. Y. Jadhav , H. N. Chaugule , S. A. Ghorpade , et al., “Design, Synthesis and Biological Evaluation of Imidazo[2,1‐*b*]thiazole–Piperazinyl Acetamide Derivatives as Potential Anticancer Agents,” Journal of Molecular Structure 1372 (2026): 146639.

[17] D. Zolotareva , A. Zazybin , A. Dauletbakov , et al., “Morpholine, Piperazine, and Piperidine Derivatives as Antidiabetic Agents,” Molecules 29, no. 13 (2024): 3043. 10.3390/molecules29133043 PMC11243478 38998996

[18] A. Nagani , M. Shah , S. Patel , et al., “Unveiling Piperazine‐Quinoline Hybrids as Potential Multi‐Target Directed Anti‐Alzheimer's Agents: Design, Synthesis and Biological Evaluation,” Molecular Diversity 29, no. 2 (2025): 1453–1478. 10.1007/s11030-024-10927-4 38990393

[19] C. Biba , I. Varasi , P. A. Cavallaro , et al., “P‐216 Evaluation of Broad‐Spectrum Piperazine‐Based Compounds Able to Inhibit Flavivirus and/or SARS‐CoV‐2 Replication in a Live Virus Assay,” Sexually Transmitted Infections 100 (2024): A273–A274, 10.1136/sextrans-ICAR-2024.269.

[20] X. Jiang , B. Huang , S. Rumrill , et al., “Discovery of Diarylpyrimidine Derivatives Bearing Piperazine Sulfonyl as Potent HIV‐1 Nonnucleoside Reverse Transcriptase Inhibitors,” Communications Chemistry 6, no. 1 (2023): 83. 10.1038/s42004-023-00888-4 PMC10148624 37120482

[21] M. Al‐Ghorbani , M. A. Gouda , M. Baashen , O. Alharbi , F. A. Almalki , and L. V. Ranganatha , “Piperazine Heterocycles as Potential Anticancer Agents: A Review,” Pharmaceutical Chemistry Journal 56, no. 1 (2022): 29–37.

[22] Y. Wang , K. Xu , G. Bai , et al., “Synthesis and Antifungal Activity of Novel Triazole Compounds Containing Piperazine Moiety,” Molecules 19, no. 8 (2014): 11333–11340. 10.3390/molecules190811333 PMC6271785 25090121

[23] M. Somashekhar and A. R. Mahesh , “Synthesis and Antimicrobial Activity of Piperazine Derivatives,” American Journal of PharmTech Research 3, no. 4 (2013): 640–645.

[24] L. D. Roy , S. Remady , and J. Kumar , “Piperazine Tethered Heterocyclic Hybrids: Synthesis, Characterization, and In Vitro Studies From Potent Proteinase Inhibitors to Antiproliferative Activity,” European Journal of Medicinal Chemistry Reports 10 (2024): 100132.

[25] S. Mahammad Ghouse , K. Bahatam , A. Angeli , et al., “Synthesis and Biological Evaluation of New 3‐Substituted Coumarin Derivatives as Selective Inhibitors of Human Carbonic Anhydrase IX and XII,” Journal of Enzyme Inhibition and Medicinal Chemistry 38, no. 1 (2023): 2185760. 10.1080/14756366.2023.2185760 PMC10013565 36876597

[26] S. Aggarwal , D. Jain , P. Kaur , and K. Singh , “Advances in Piperazine‐Based Compounds for Antimicrobial Drug Development: Design, SAR, and Therapeutic Potential,” Current Drug Research Reviews 18, no. 2 (2025): 235–247. 10.2174/0125899775378431250611100229 40873355

[27] A. T. King , L. Matesic , S. T. Keaveney , and J. F. Jamie , “An Investigation Into the In Vitro Metabolic Stability of Aryl Sulfonyl Fluorides for Their Application in Medicinal Chemistry and Radiochemistry,” Molecular Pharmaceutics 20, no. 2 (2023): 1061–1071. 10.1021/acs.molpharmaceut.2c00806 36638322

[28] T. I. Patel , M. L. Williams , J. M. Linder , S. S. Shah , and M. J. Moschitto , “Sulfonylation of Anilines Through Photocatalytic Activation of *N*‐Hydroxymethylphthalimide Sulfones,” ACS Catalysis 16, no. 1 (2025): 744–751.

[29] Z. M. Lai , Y. Xie , L. L. Huang , J. Guo , and G. Lu , “Catalytic Enantioselective Synthesis of α‐C Chiral Sulfones Enabled by Merging Photoactive Electron Donor–Acceptor Complexes With Nickel Catalysis,” Chemical Science 16, no. 10 (2025): 4352–4359. 10.1039/d4sc07264b PMC11793018 39911333

[30] P. S. Phatak , R. D. Bakale , R. S. Kulkarni , et al., “Design and Synthesis of New Indanol‐1,2,3‐Triazole Derivatives as Potent Antitubercular and Antimicrobial Agents,” Bioorganic & Medicinal Chemistry Letters 30, no. 22 (2020): 127579. 10.1016/j.bmcl.2020.127579 32987135

[31] U. V. Mhetre , N. B. Haval , G. M. Bondle , et al., “Design, Synthesis and Molecular Docking Study of Novel Triazole–Quinazolinone Hybrids as Antimalarial and Antitubercular Agents,” Bioorganic & Medicinal Chemistry Letters 108 (2024): 129800. 10.1016/j.bmcl.2024.129800 38763480

[32] E. P. Amaral , C. L. Vinhaes , D. Oliveira‐de‐Souza , B. Nogueira , K. M. Akrami , and B. B. Andrade , “The Interplay Between Systemic Inflammation, Oxidative Stress, and Tissue Remodeling in Tuberculosis,” Antioxidants & Redox Signaling 34, no. 6 (2021): 471–485. 10.1089/ars.2020.8124 PMC8020551 32559410

[33] A. Reichel and P. Lienau , “Pharmacokinetics in Drug Discovery: An Exposure‐Centred Approach to Optimising and Predicting Drug Efficacy and Safety,” in New Approaches to Drug Discovery (Springer, 2015), 235–260. 10.1007/164_2015_26 26330260

[34] J. A. Pradeepkiran , S. B. Sainath , and K. V. Shrikanya , “In Silico Validation and ADMET Analysis for the Best Lead Molecules,” in Brucella melitensis (Academic Press, 2021), 133–176.

[35] R. D. Bakale , P. S. Phatak , S. S. Rathod , et al., “In Vitro and In Silico Exploration of Newly Synthesized Triazolyl‐Isonicotinohydrazides as Potent Antitubercular Agents,” Journal of Biomolecular Structure and Dynamics 43, no. 3 (2025): 1372–1391. 10.1080/07391102.2023.2291826 38079301

[36] R. D. Bakale , S. M. Sulakhe , S. L. Kasare , et al., “Design, Synthesis and Antitubercular Assessment of 1,2,3‐Triazole Incorporated Thiazolylcarboxylate Derivatives,” Bioorganic & Medicinal Chemistry Letters 97 (2024): 129551. 10.1016/j.bmcl.2023.129551 37979730

[37] M. Reddy Gangireddy , M. Mantipally , R. Gundla , V. Nayak Badavath , K. Paidikondala , and A. Yamala , “Design and Synthesis of Piperazine‐Linked Imidazo[1,2‐*a*]pyridine Derivatives as Potent Anticancer Agents,” ChemistrySelect 4, no. 46 (2019): 13622–13629.

[38] C. Li , X. Tian , Z. Huang , et al., “Structure–Activity Relationship of Novel Pyrimidine Derivatives With Potent Inhibitory Activities Against *Mycobacterium tuberculosis* ,” Journal of Medicinal Chemistry 66, no. 4 (2023): 2699–2716. 10.1021/acs.jmedchem.2c01647 36735271

[39] S. A. Shaikh , S. R. Labhade , R. R. Kale , et al., “Thiadiazole‐Thiazole Derivatives as Potent Anti‐Tubercular Agents: Synthesis, Biological Evaluation, and In Silico Docking Studies,” European Journal of Medicinal Chemistry Reports 12 (2024): 100183.

[40] S. A. Shaikh , S. R. Labhade , R. R. Kale , et al., “Synthesis, Biological and Molecular Docking Studies of Thiazole‐Thiadiazole Derivatives as Potential Anti‐Tuberculosis Agents,” Chemistry & Biodiversity 21, no. 6 (2024): e202400496. 10.1002/cbdv.202400496 38700369

[41] S. A. Ghorpade , A. S. Kulkarni , P. B. Dandge , et al., “Methoxy Substituted Furan Chalcones: An Insight Into Anti‐Inflammatory, Antioxidant, Antidiabetic, Antibacterial, and Molecular Docking Studies,” ChemistrySelect 11, no. 11 (2026): e05798.

[42] A. Yadava , M. M. R. Bhuyan , D. Mukherjee , D. Kumar , and M. Dwivedi , “Phytomolecules as Potential Candidates to Intervene the Function of *E. coli* Sodium‐Proton Antiporters; Ec‐NhaA,” Journal of Biomolecular Structure and Dynamics 41, no. 24 (2023): 15598–15609. 10.1080/07391102.2023.2191130 36935099

[43] V. Goswami , D. Patel , S. Rohit , U. Chaube , and B. Patel , “Homology Modeling, Binding Site Identification, Molecular Docking and Molecular Dynamics Simulation Study of Emerging and Promising Drug Target of Wnt Signaling–Human Porcupine Enzyme,” Results in Chemistry 7 (2024): 101482.

[44] P. K. Deb , N. A. Al‐Shar'i , K. N. Venugopala , M. Pillay , and P. Borah , “In Vitro Anti‐TB Properties, In Silico Target Validation, Molecular Docking and Dynamics Studies of Substituted 1,2,4‐Oxadiazole Analogues Against *Mycobacterium tuberculosis* ,” Journal of Enzyme Inhibition and Medicinal Chemistry 36, no. 1 (2021): 869–884. 10.1080/14756366.2021.1900162 PMC8172222 34060396

[45] N. A. Peerzade , S. Y. Jadhav , R. B. Bhosale , et al., “Pyrazole‐Based *N*‐Phenyl Pyrazolines: Synthesis, Docking, and Pharmacological Evaluation,” Results in Chemistry 11 (2024): 101793.

[46] S. Singha , S. Agrawal , A. Kumar , H. Abbas , A. Kumar , and V. Ranjan , “Computational Pharmacology and Pharmacokinetics of Betanin: An Integrated ADMET, SwissADME, DFT, and Target Prediction Study,” Journal of Pharmaceutical Sciences 1, no. 11 (2025): 190–203.

