# Supplementary material for: Structure‐Based Design and Biological Evaluation of Imidazo[1,2‐a]pyridine Derivatives as Potential Antituberculosis Agents
Source: Chem Biodivers. 2026 Oct 22;23(10):e71854. doi: 10.1002/cbdv.71854 (PMC13691380; doi:10.1002/cbdv.71854)
Supplement: Supplementary file 1 [file CBDV-23-e71854-s001.docx]

**Supporting Information**

| **Sr. No** | **Description** |
| --- | --- |
| 1 | NMR Spectra for **compounds** **(4a-4k)** (Figure S1 to Figure S22) |
| 2 | FT-IR Spectra for Compounds (**4a-4k)** (Figure S23 to Figure S33) |
| 3 | HRMS Spectra for Representative Compounds **(4a-4k)** (Figure S34 to Figure S43) |
| 4 | General Procedure for Antioxidant Activity |
| 5 | General Procedure for Haemolytic Activity |

NMR Spectra for compounds **(4a-4k)**

Figure S1: ^1^H-NMR spectrum of compound **(4a)** in CDCl_3_

Figure S2: ^13^C-NMR spectrum of compound **(4a)** in CDCl_3_

Figure S3: ^1^H-NMR spectrum of compound **(4b)** in CDCl_3_

Figure S4: ^13^C-NMR spectrum of compound **(4b)** in CDCl_3_

Figure S5: ^1^H-NMR spectrum of compound **(4c)** in CDCl_3_

Figure S6: ^13^C-NMR spectrum of compound **(4c)** in CDCl_3_

Figure S7: ^1^H-NMR spectrum of compound **(4d)** in CDCl_3_

Figure S8: ^13^C-NMR spectrum of compound **(4d)** in CDCl_3_

Figure S9: ^1^H-NMR spectrum of compound **(4e)** in CDCl_3_

Figure S10: ^13^C-NMR spectrum of compound **(4e)** in CDCl_3_

Figure S11: ^1^H-NMR spectrum of compound **(4f)** in CDCl_3_

Figure S12: ^13^C-NMR spectrum of compound (**4f)** in CDCl_3_

Figure S13: ^1^H-NMR spectrum of compound **(4g)** in CDCl_3_

Figure S14: ^13^C-NMR spectrum of compound (**4g)** in CDCl_3_

Figure S15: ^1^H-NMR spectrum of compound **(4h)** in CDCl_3_

Figure S16: ^13^C-NMR spectrum of compound (**4h)** in CDCl_3_

Figure S17: ^1^H-NMR spectrum of compound **(4i)** in CDCl_3_

Figure S18: ^13^C-NMR spectrum of compound (**4i)** in CDCl_3_

Figure S19: ^1^H-NMR spectrum of compound **(4j)** in CDCl_3_

Figure S20: ^13^C-NMR spectrum of compound (**4j)** in CDCl_3_

Figure S21: ^1^H-NMR spectrum of compound **(4k)** in CDCl_3_

Figure S22: ^13^C-NMR spectrum of compound (**4k)** in CDCl_3_

IR Spectra for Representative Compounds **(4a-4k)**

Figure S23: IR spectrum of compound **4a**

Figure S24: IR spectrum of compound **4b**

Figure S25: IR spectrum of compound **4c**

Figure S26: IR spectrum of compound **4d**

Figure S27: IR spectrum of compound **4e**

Figure S28: IR spectrum of compound **4f**

Figure S29: IR spectrum of compound **4g**

Figure S30: IR spectrum of compound **4h**

Figure S31: IR spectrum of compound **4i**

Figure S32: IR spectrum of compound **4j**

Figure S33: IR spectrum of compound **4k**

Mass Spectra for Representative Compounds **(4a-4k)**

Figure S34: HRMS of compound **4a**

Figure S35: HRMS of compound **4b**

Figure S36: HRMS of compound **4c**

Figure S37: HRMS of compound **4e**

Figure S38: HRMS of compound **4f**

Figure S39: HRMS of compound **4g**

Figure S40: HRMS of compound **4h**

Figure S41: HRMS of compound **4i**

Figure S42: HRMS of compound **4j**

Figure S43: HRMS of compound **4k**

**2.4 General Procedure for Antioxidant Activity**

The antioxidant activity of tested compounds (**4a-4k**) evaluated by reported 2, 2-diphenyl-1-picrylhydrazyl radical scavenging assay (DPPH) and Hydroxyl radical assay [1].

**DPPH Assay**

The antelectron transfer ability of the synthesized compounds was measured from the bleaching of the purple-coloured methanolic solution of DPPH. Equal volume of the compound and DPPH solution in methanol were mixed so that the final volume of reached 3 ml. Next, incubated the samples for 20 minutes at R.T. and measured the absorbance at 517 nm. 1 mM ascorbic acid was used as a standard. The inhibition percentage was calculated using the following formula:

w% radical scavenging activity = 1 – T/C x 100

**OH radical Assay**

The Fenton reaction demonstrated the (OH) radical activity. The reaction mixture of 90µL of 1-10 phenanthroline (1 mM), 60µL of FeCl_2_ (1 mM) and 150 µL of H_2_O_2_ (0.17 M), 2.4 mL of phosphate buffer (0.2 M, pH 7.8) and 1.5 mL of individual synthesized compound (1 mg/mL) were mixed. The reaction was initiated by adding hydrogen peroxide. After 5 minutes of incubation at R.T., the absorbance was measured at 560 nm. Ascorbic acid (1 mM) was used as a reference standard.

% radical scavenging activity = 1 – T/C x 100

**2.5 General Procedure Haemolytic Activity**

The haemolytic activities using human RBCs for the selected synthesized compounds were determined [2]. Human blood (5 mL) was initially collected in tubes containing 1mg of EDTA (anti-coagulant). The erythrocytes were collected by centrifugation at 2000 rpm at 20 ^°^C for 10 minutes. The collected pellet was washed thrice with PBS. Then, 10 % (v/v) erythrocytes/PBS suspension was made using PBS1-6. From this, 1:10 dilution was achieved using PBS, which was used for the assay. 100 µL of erythrocytes were added to each well containing synthesized compounds 100 µL (500 µg/mL). 0.001N Triton X 100 was used as a reference 5. The tubes were incubated for 1 hr. at 37 ºC and centrifuged repeatedly. From the supernatant, 150 µL solution was transferred to 96 well microplates, and the absorbance was measured at 540 nm using a plate reader. 7% haemolysis was calculated using the following equation:

Haemolysis= [(A_540 of test compound sample-A_540 of buffered sample) ÷ (A_540 of Triton X 100 treated sample-A_540 buffered sample)] ×100

**Statistical analysis:** All the biological experiments were conducted in the triplicate and the results are shown to be the mean values of three independent experiments. Antioxidants activities were shown to be n=3, ±SD.

**References:**

[1] Shaikh SA, Labhade SR, Kale RR, Pachorkar PY, Meshram RJ, Jain KS, Labhade HS, Bhanushali DD, More RA, Nerkar CK, Chobe SS. Thiadiazole-thiazole derivatives as potent anti-tubercular agents: Synthesis, biological evaluation, and *In silico* docking studies. European Journal of Medicinal Chemistry Reports. 2024 Dec 1;12:100183.

[2] Shaikh SA, Labhade SR, Kale RR, Pachorkar PY, Meshram RJ, Jain KS, Labhade HS, Boraste DR, More RA, Chobe SS, Ballabh D. Synthesis, biological and molecular docking studies of thiazole‐thiadiazole derivatives as potential anti‐tuberculosis agents. Chemistry & Biodiversity. 2024 Jun;21(6):e202400496.
